# Characterization and application of recombinant Bovine Leukemia Virus Env protein

**DOI:** 10.1038/s41598-024-62811-8

**Published:** 2024-05-28

**Authors:** Lorena Tomé-Poderti, Natalia Olivero-Deibe, Federico Carrión, María Magdalena Portela, Gonzalo Obal, Gleysin Cabrera, Sergio Bianchi, Analia Lima, Andrés Addiego, Rosario Durán, Gonzalo Moratorio, Otto Pritsch

**Affiliations:** 1 Immunovirology Lab, Institut Pasteur de Montevideo, 11400 Montevideo, Uruguay; 2 Analytical Biochemistry and Proteomics Unit, Instituto de Investigaciones Biológicas Clemente Estable/Institut Pasteur de Montevideo, 11400 Montevideo, Uruguay; 3 Facultad de Ciencias, Universidad de la República, 11400 Montevideo, Uruguay; 4 Laboratory of Molecular Biomarkers, Department of Physiopathology, University Hospital, Universidad de la República, 11600 Montevideo, Uruguay; 5 Functional Genomics Unit, Institut Pasteur de Montevideo, 11400 Montevideo, Uruguay; 6 Experimental Evolution of Viruses, Institut Pasteur de Montevideo, 11400 Montevideo, Uruguay; 7 Laboratorio de Virología Molecular, Facultad de Ciencias, Universidad de la República, Montevideo, Uruguay; 8 Immunobiology Department School of Medicine, Universidad de la República, 11800 Montevideo, Uruguay; 9 Present Address: Morphogenesis and Antigenicity of HIV and Hepatitis Viruses (MAVIVH), INSERM Unit 1259, Université de Tours and CHRU de Tours, Tours, France

**Keywords:** Biochemistry, Immunology, Microbiology

## Abstract

The Bovine Leukemia Virus (BLV) Envelope (Env) glycoprotein complex is instrumental in viral infectivity and shapes the host’s immune response. This study presents the production and characterization of a soluble furin-mutated BLV Env ectodomain (sBLV-EnvFm) expressed in a stable S2 insect cell line. We purified a 63 kDa soluble protein, corresponding to the monomeric sBLV-EnvFm, which predominantly presented oligomannose and paucimannose *N-*glycans, with a high content of core fucose structures. Our results demonstrate that our recombinant protein can be recognized from specific antibodies in BLV infected cattle, suggesting its potential as a powerful diagnostic tool. Moreover, the robust humoral immune response it elicited in mice shows its potential contribution to the development of subunit-based vaccines against BLV.

## Introduction

Bovine Leukemia Virus (BLV) is a B-lymphotropic oncogenic retrovirus of the genus *Deltaretrovirus* that infects mainly dairy cattle worldwide and is the causative agent of enzootic bovine leukosis (EBL)^1,2^. Approximately 70% of infected cattle remain asymptomatic which explains why BLV has been neglected in several regions worldwide, accounting for an extremely high prevalence (30–90%) and imposing a severe economic impact on the dairy cattle industry^3^. While asymptomatic carriers never show haematological signs of infection, one third of BLV-infected animals will develop persistent lymphocytosis characterized by a non-malignant polyclonal expansion of CD5 + B-cells. Only 5–10% of infected cows will develop malignant monoclonal B-cell lymphosarcoma after long latency periods (4–10 years)^4–6^.

Propagation of BLV occurs via vertical and horizontal transmission^7,8^. In the past, countries from West Europe and Oceania have eradicated BLV by implementing control and eradication programs, based on “test and culling”. However, this strategy remains infeasible in the Americas and other countries from Eastern Europe and Asia where EBL is endemic, showing extremely high prevalence rates (up to 90% of BLV-positive dairy cattle)^9^. In the absence of commercially available vaccines or effective treatments, one of the most successful measures to counteract BLV infection and propagation is based on reduction of transmission via “test and segregate”, with the aim of removing animals with the highest proviral loads and lymphocytosis. These animals would be mainly responsible for a high viral transmissibility at the farm level, and their selective elimination would have a relevant impact on reducing the infection of healthy livestock^10^.

As in all retroviruses, the BLV *env* gene codes for a polyprotein precursor (pr72)^11^, whose N-terminus is generated by signalase cleavage in the endoplasmic reticulum of the infected cell. During its transport to the budding site, pr72 is cleaved by furin-like endoproteases into subunits gp51 (or surface subunit, N-terminal portion) and gp30 (or transmembrane subunit, C-terminal portion), which are kept linked by disulfide bonds (characteristic shared by alpha, gamma and delta retroviruses)^12,13^. In BLV, gp51-gp30 association in the prefusion conformation is stabilized by disulfide bonds between C_212_XXC_215_ in gp51 and _384_CX_6_CC_392_ in gp30 (being X any amino acid)^14^. Upon processing, disulfide-linked gp51-gp30 subunits keep attached to the membrane as homotrimeric complexes known as BLV Env glycoprotein (gp51/gp30)_3_. This (gp51/gp30)_3_ complex mediates viral entry by receptor attachment (via gp51)^15,16^ and membrane fusion (mediated by gp30) in a sequential manner^17^. Interactions with the cognate receptor^18^ trigger a conformational change in this complex, in which gp30 rearranges forcing the cellular and viral membranes, to move against each other, during membrane fusion process. Env conformational change process also involves gp51–gp30 covalent bond disruption driven by intersubunit disulfide bond isomerization to form intrasubunit S–S bonds (being C_212_ the promoter of isomerization)^19^. This precludes an early activation of fusion activity of gp30 being a process tightly regulated.

Gp51 is the most immunogenic subunit of Env and induces massive expression of specific antibodies in infected animals^20,21^. The N-terminal region of gp51 (the first 173 aa^22^) contains putative receptor binding domain (RBD^16^) together with conformational epitopes namely F, G^23^ and H, involved in infectivity and syncytia formation and two neutralization domains^23–28^. In addition, recognition of these epitopes by infected cattle sera or specific monoclonal antibodies (mAbs) depends on gp51 glycosylation^29^. This conformational epitope region is followed by a GYPD sequence (conserved in oncogenic retroviruses)^30^ and a 23aa proline rich region (PRR^16^), which delimits N and C terminal gp51 modular organization. Correspondingly, the C-terminal domain of gp51 contains linear epitopes A, B, D and E^31^.

Besides, gp30 subunit contains an extracellular N-terminal domain consisting of: (i) the hydrophobic fusion peptide, (ii) a 4–3 heptad repeat, (iii) an immunosupressor domain^32^ and (iv) the conserved _384_CX_6_CC_392_ motif that interacts with gp51 as explained above^13^. This subunit anchors the viral envelope via a transmembrane domain (aa 438–456) followed by a cytoplasmic tail (C-terminal, aa 457–515).

Like other retroviral envelope proteins, the BLV Env complex is N-glycosylated. The role of N-glycosylation of viral proteins in the stability, antigenicity and host cell invasion process has been well documented^33,34^. Modifications in the glycosylation pattern or ablation of glycosylation sites affects interaction of viral proteins with cell receptors, cell-to-cell fusion, viral replication and infectivity. Glycans associated to envelope proteins also act as a shield that confers resistance to neutralizing antibodies promoting viral immune evasion^35,36^.

**Figure 1:**
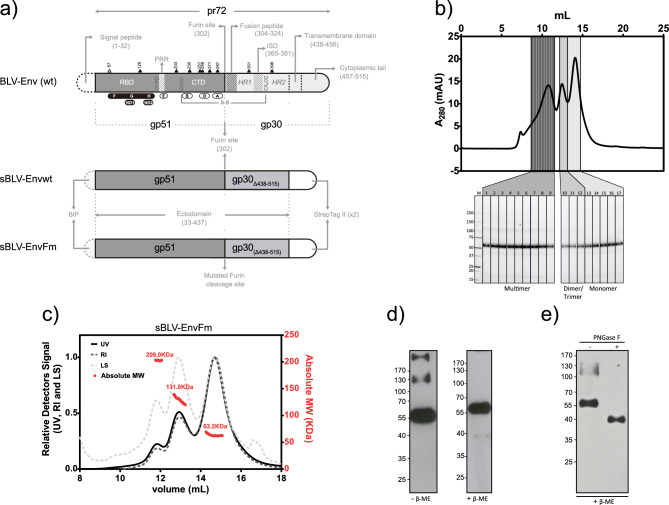
Expression and biochemical/biophysical characterization of recombinant sBLV-EnvFm protein. (**a**) Schematic representation of BLV envelope proteins both native, BLV-Env (*wt*, pr72), and soluble recombinants sBLV-Env*wt* and sBLV-EnvFm (furin-mutated). Different functional domains are indicated: signal peptide (aa 1–32 in dotted black line) replaced by the BiP secretion signal (dotted grey line) in sBLV-Env*wt* and sBLV-EnvFm; gp51 subunit (aa 33–302 in dark grey) containing the receptor binding domain (RBD), conformational (F, G and H) and linear (E, B, D, A) epitopes, the neutralization domains (ND) 1 and 2, CXXC motif for disulfide bond, GYPD and PRR domains; furin cleavage site (aa 302) or mutated furin cleavage site (in recombinant protein aa 300–301) are indicated with arrows; gp30 subunit (aa 303–515 in light grey) containing the fusion peptide, the heptad repeat domains (HR) 1 and 2, the immunosuppression domain (ISD), the CX_6_CC disulfide bond motif and the transmembrane domain which divides the ectodomain (aa 33–437) from the cytoplasmic portion and anchors the BLV-Env *wt* protein into lipid bilayers. In recombinant sBLV-Env and sBLV-EnvFm proteins, aa 438–515 where replaced by a double strepTag II (white). Potential N-linked glycosylation sites N67, N129, N203, N230, N251, N256, N271, N287, N351, N398 are shown (black triangles: predicted N-glycosylated sites, white triangles: not predicted sequons). (**b**) Superdex 200 SEC analysis of sBLV-EnvFm purified by affinity cromathography (top) with non-reducing SDS-PAGE fraction analysis (bottom). Grey scale-colored fractions indicate multimeric (fractions 1–9), dimeric/trimeric (fractions 10–12) and monomeric (fractions 13–17) forms of the protein. A 55 kDa band corresponding to sBLV-EnvFm is present in all fractions tested. (**c**) Multi-angle static light scattering coupled to size exclusion chromatography (SEC-MALS) of monomeric sBLV-EnvFm (fractions 13–17 in panel **b**). Absorbance at 280 nm (UV), refractive index (RI) and light scattering at 90° angle (LS) are shown overlaid with absolute molecular weights (red scattered dots with average MW in kDa). As shown sBLV-EnvFm is mainly eluted as monomer (63.2 kDa) together with dimer/trimer oligomers. (**d**) Western blot analysis of purified sBLV-EnvFm under reducing (+ β-ME) and non-reducing (− β-ME) conditions using anti-gp51 mAb showing that the presence of high molecular weight oligomers corresponding to 130 and 200 kDa can be due to S–S interactions. (**e**) Enzymatic deglycosylation of sBLV-EnvFm with PNGase F (+) in reducing conditions (+ β-ME). Non-deglycosylated protein was included as control, PNGase F (−). A precise mobility shift of 10 kDa between deglycosylated sBLV-EnvFm vs non-deglycosylated sBLV-EnvFm was observed. Gels and blots were cropped to improve clarity, original gels/blots are presented in Supplementary Fig. S11. *aa* amino acid, *kDa* kilodaltons, *mAU* milliabsorbance units, *β-ME* β-mercaptoethanol.

In this regard BLV Env protein contains 10 putative asparagine (N)-linked glycosylation sites unevenly distributed between both subunits, with gp51 harboring the majority of the putative N-glycosylation sites (Fig. 1a).

First studies on BLV Env N-glycosylation, were mainly focused on the development of strategies to analyze gp51 glycosylation by pharmacological inhibition of different glycosylation pathways, the interference with glycan binding and site directed mutagenesis of N-glycosyation potential sites in infectious provirus^37,38^. Later, de Brogniez et al.demonstrated that single mutations at different potential glycosylation sites in BLV-gp51 neither ablated protein functionality nor introduced “apparent” structural modifications as they didn’t inhibit in vitro cell fusion or in vivo infectivity^37^. Contrarily, simultaneous mutation of these potential glycosylation sites revoked infectivity in vivo consistent with a role in the viral persistence or replication^37^. From this and other studies focused on gp51, only N129, N203, N230, N251, N256 and N271 were found to be glycosylated^37,39–41^. Hence, it is imperative to conduct a comprehensive analysis of all potential N-glycosites within the ectodomain of the BLV Env protein (gp51–gp30).

Recently, Olivero et al. have demonstrated that *Drosophila* S2 cells co-transfected with the *env* and *gag* sequences were able to form virus-like particles (VLPs) displaying a BLV Env which retains structural motifs on its surface^42^. Stably transfected *Drosophila melanogaster* S2 cells have appeared as a major platform for recombinant protein expression^43,44^. N-glycosylation in S2 insect cells gives oligomannosidic or paucimannosidic glycans with lower complexity than mammalian cells^45^, making it a suitable eukaryotic expression system for biophysical, biochemical and structural characterization of recombinant secreted viral glycoproteins, as well documented in Carrión et al. and others^46,47^. Furthermore, S2 cells are currently being utilized for the production of potential vaccine antigens, demonstrating excellent performance in inducing neutralizing antibodies, as recently evidenced in the production of the Zika E viral protein^48^. Consequently, the involvement of non-complex *N-*glycans paves the way for the refinement of subunit vaccine candidates, leveraging the potential of immunogens produced in insect cells.

Here, we report the production and characterization of a soluble furin-mutated BLV Env ectodomain (sBLV-EnvFm) expressed in a stable S2 insect cell line. A soluble protein of 63 kDa corresponding to monomeric sBLV-EnvFm was purified, presenting oligomannose and paucimannose *N-*glycans (with high content of core fucose structures) as major glycoforms. Our results demonstrate that our recombinant protein can be recognized by specific antibodies in BLV infected cattle suggesting that it could be used as a powerful diagnosis tool. Moreover, the robust humoral immune response it elicited in mice shows its potential contribution to the development of subunit-based vaccines against BLV.

## Results

### Recombinant soluble BLV-Env*wt* and BLV-EnvFm proteins were expressed in Drosophila S2 cells

Soluble *wild type* (sBLV-Env*wt*) and furin-mutated (sBLV-EnvFm) ectodomains of BLV-Env (Fig. 1a) were expressed in Drosophila S2 cells. Both proteins were purified by affinity chromatography 30 h post induction, showing similar purification yields (11 mg L^−1^). Secreted proteins evidenced signs of degradation at 48 h post induction as observed with sBLV-EnvFm (Supplementary Fig. S1).

sBLV-Env*wt* purification by size exclusion chromatography (SEC) showed it was composed mainly by high molecular weight (HMW) aggregates, with a small fraction containing low molecular weight (LMW) species. SDS-PAGE analysis showed that both fractions were composed mainly by two bands of ~ 40 kDa and ~ 20 kDa corresponding to gp51 and gp30-ectodomain sizes (Supplementary Fig. S2a,b). Bands corresponding to full-length non-cleaved ectodomain were only detected in HMW fraction, accompanied by other proteins of variable sizes. Anti-gp51 western blot, evidenced the presence of a band > 35 kDa accompanied by a less abundant band of ~ 55 kDa (as expected for gp51 and non-cleaved Env ectodomain, respectively) (Supplementary Fig. S2c). However, gp51-containing products were detected throughout the entire affinity purification process, suggesting its dissociation from the Strep-tag-containing gp30 subunit.

On the other hand, sBLV-EnvFm was subjected to similar analysis using SEC and non-reducing SDS-PAGE. This revealed a single band greater than 50 kDa, corresponding to the non-proteolyzed full-length recombinant protein. The protein exhibited various oligomerization states, including monomer (less than 75 kDa), dimer/trimer (120–180 kDa), and higher order soluble multimers (greater than 440 kDa) (Fig. 1b). Monomeric fraction was further characterized by SEC-MALS showing that the majority of the protein was kept in its monomeric state with an absolute molecular weight of 63.2 kDa, accompanied by different size oligomers mainly corresponding to dimers and trimers of 131 and 209 kDa, respectively (Fig. 1c). DLS analysis of sBLV-ENVFm showed a polydisperse Intensity distribution (PdI = 0.531 ± 0.10) with two size populations: a principal peak with Hydrodynamic Radii, RH = 6.5 ± 0.2 nm and a minority peak with RH = 95.2 ± 1.4 nm (Supplementary Fig. S3).

These results are in agreement with anti-gp51 western blot analysis under non-reducing conditions, evidencing a band of > 55 kDa accompanied by two additional bands of 130 kDa and > 170 kDa (Fig. 1d). These additional bands were not detected under reducing conditions suggesting a potential contribution of disulfide bonds in oligomer and aggregate formation. Considering the higher stability evidenced by the proteolytic processing defective mutant sBLV-EnvFm, with respect to sBLV-Env*wt*, we decided to focus our work on sBLV-EnvFm.

The observed discrepancies between the predicted mass of sBLV-EnvFm monomers (49.8 kDa) and the absolute molecular weight measured by SEC-MALS (63.2 kDa) are likely due to N-glycosylation. To validate this hypothesis, we performed a western blot analysis on sBLV-EnvFm digested with endoglycosidase PNGase F. This revealed a shift of over 10 kDa, further supporting our hypothesis (Fig. 1e).

### *N-*glycan profiling analysis of sBLV-EnvFm reveals fucosylated pauci/oligo mannose as the main glycan component

**Figure 2:**
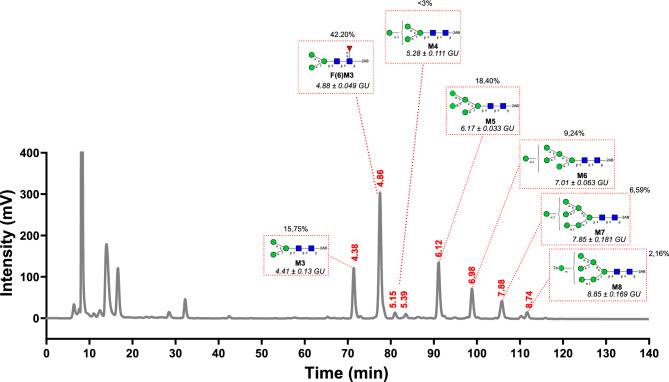
*N*-glycan content of sBLV-EnvFm obtained by normal-phase (NP) HPLC. Released *N*-glycans of sBLV-EnvFm (upon PNGase F treatment and derivatization with 2AB) were purified by NP-HPLC. GU values are assigned based on retention times (min) of each fraction in comparison with a dextran hydrolysate analyzed under the same HPLC separation conditions. Quantitative estimation of glycan content in this protein was calculated by measuring the ratios of peak areas (as a percentage of total glycans). Relative abundance of each glycan molecular form was calculated from peak area of NP-HPLC. Experimental values were compared with the GlycoStore database (http://www.glycostore.org) for the assignment of possible structures linked to the BLV Env glycoprotein (Supplementary Table S1). *N*-glycan species were assigned based on the Glycostore, upon refinement with exoglycosydase digestion results (Supplementary Fig. S4) and the major *N*-glycan structures described for insects. Most probable glycan structures are shown in dotted red boxes next to each peak, as cartoon diagrams with its Oxford notation and Glycostore GU (± s.d). Symbolic representation of glycans is as follows: GlcNAc, filled blue square; Man, filled green circle; fucose, filled red triangle; beta (β) linkage (1–2 or 1–4), solid line; alpha (α) linkage, solid line 1–3 linkage, (/), 1–6 linkage, (\), horizontal line 1–2 linkage; vertical solid line, fucose α1,6-linked to the inner GlcNAc. *GU* glucose units, *2AB* 2-aminobenzamide, *GlcNAc* β-*N*-acetylglucosamine, *Man* mannose, *Fuc* fucose.

Glycan moieties attached to sBLV-EnvFm were characterized by normal phase (NP) HPLC, exoglycosidase treatment and MALDI –TOF mass spectrometry. PNGase F-glycan release followed by 2AB labelling and separation on normal phase HPLC was performed to analyze composition of *N-*glycans in sBLV-EnvFm. Retention times of each fraction were expressed in glucose units (GU) and experimental values were compared with theoretical GU values reported in the GlycoStore database (www.glycostore.org) for the assignment of possible structures linked to the BLV Env glycoprotein (Supplementary Fig. S4a, Supplementary Table S1). *N-*glycan structures were assigned based on GU values as described above, together with sequential exoglycosidase treatment (Supplementary Fig. S4b–f). Briefly exoglycosydase treatment showed the presence of pauci and oligomannosidic glycans with a strong presence of fucosylation and a marginal or null contribution of sialic acid (Supplementary Fig. S4b,c). Moreover, we confirmed the absence of galactose and terminal GlcNAc, since no modifications in the elution profile were detected upon digestion with BTG and GUH, respectively (Supplementary Fig. S4d,e). Finally, the high content of mannose was evidenced by JBM treatment (Supplementary Fig. S4f). MALDI-TOF MS of 2AB-derivatized glycans further confirmed the structures described above (Supplementary Fig. S5). The global interpretation of our results on sBLV-EnvFm glycan analysis is summarized in Fig. 2, showing its *N-*glycan composition and relative abundance calculated from peak area of NP-HPLC. The main type of *N-*glycan obtained in sBLV-EnvFm corresponded to fucosylated paucimannose glycan Man_3_GlcNAc_2_Fuc (F(6)M3) with 42,2% (major peak at 4,86 GU). Core Man_3_GlcNAc_2_ (M3) (initial N-glycosylation core) is present in all *N-*glycans. The rest of the structures reflect simple *N-*glycan addition of only mannose residues leading to pauci and oligomannosidic glycans (Fig. 2, Supplementary Table S1). Thus *N-*glycans with high-mannose content (Man_5-9_GlcNAc_2_) represent 36,4% of total glycans detected. These results were also confirmed in sBLV-EnvFm glycopeptides analyzed by nanoLC-MS/MS (Supplementary Tables S2, S3).

Regarding *N-*glycan positions on sBLV-EnvFm sequence, we first predicted a total of 9 potential N-linked glycosylation sites with potential scores above 0.4: 7 in gp51 (N129, N203, N230, N251, N256, N271, N287) and 2 in gp30 (N351, N398). Two N-glycosites were predicted with very low scores at N67 and N300 (Supplementary Fig. S6).

**Figure 3:**
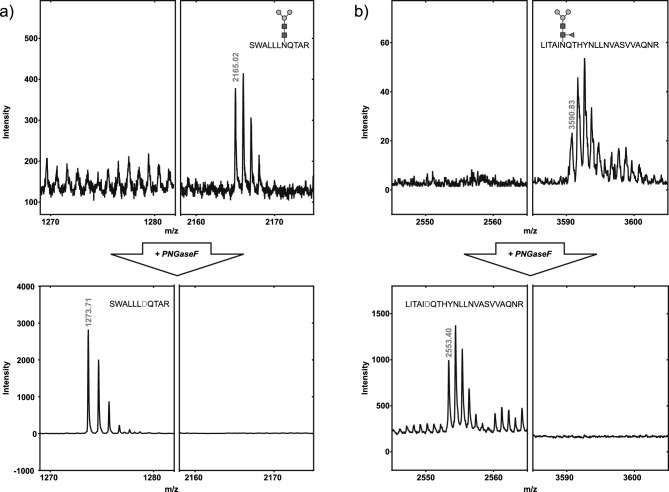
Analysis of sBLV-EnvFm glycosylated peptides by mass spectrometry. MALDI mass spectrum of tryptic peptides SWALLLNQTAR (sequence 197–207) and LITAINQTHYNLLNVASVVAQNR (sequence 346–368), identified as glycosylated peptides containing two of the most abundant glycan structures in NP-HPLC, with *m/z* experimental values of 2165.02 (**a**, top panel) and 3590.83 (**b**, top panel). Comparison with MALDI spectra obtained upon treatment with PNGase F allowed us to identify deglycosylated forms of each peptide (**a**,**b**, bottom panels). For SWALLLNQTAR (**a**) a signal of *m/z* = 1273.71 appears after PNGase F treatment, corresponding to the non-glycosylated sequence (*m/z* = 1272.71) with N replaced by D (glycosidase induced-deamination) and the peak with *m/z* = 2165.02 disappears. MS/MS analysis confirmed that this signal corresponded to glycosylated SWALLLNQTAR, with N203 showing a mass increment of 892,3 compatible with the incorporation of Man_3_GlcNAc_2_ (Supplementary Supplementary Fig. S6a). Similarly, for LITAINQTHYNLLNVASVVAQNR (**b**) we detect a signal of *m/z* of 2553.4 only after deglycosylation, (theoretical *m/z* = 2552.39), indicating the presence of one glycosylation site. At the same time, a peak corresponding to the single charged peptide plus Man_3_GlcNAc_2_Fuc (1039 Da), clearly detected in the glycosylated form, disappears after PNGase F treatment. As before MS/MS analysis confirmed the sequence of the glycopeptide and N351 as the glycosylation site (Supplementary Fig. S6b). Structure abbreviations: *M3* Man_3_GlcNAc_2_, *F(6)M3* Man_3_GlcNAc_2_Fuc, *GlcNAc* β-*N*-acetylglucosamine, *Man* mannose, *Fuc* fucose.

MALDI-TOF MS analysis of sBLV-EnvFm before and after treatment with PNGase F (causing deamidation of asparagines upon glycan removal^49,50^) allowed preliminary identification of putative N-glycosites (Supplementary Table S4), as seen for two glycopeptides in Fig. 3 (SWALLLNQTAR and LITAINQTHYNLLNVASVVAQNR, containing two of the most abundant moieties M3 and F(6)M3, respectively). MS/MS analysis of these peptides further confirmed the glycosylation and showed that N203 and N351 were the modified residues as expected (Supplementary Fig. S7). This strategy allowed the identification of all nine predicted sites: N129, N203, N230, N251, N256, N271, N287, N351 and N398 which were further confirmed by nanoLC-MS/MS (except for N129 that was only detected as glycosylated by MALDI-TOF after PNGase F digestion) (Supplementary Tables S2, S3 and S4). N67 was exclusively found as non-glycosylated (by MALDI-TOF and nanoLC-MS/MS) confirming that this is not a N-glycosite.

Despite glycosylation at N300 was predicted with a very low score (Supplementary Fig. S6) and not found in MALDI-TOF analysis (Supplementary Table S4), our nanoLC-MS/MS results show that substitutions introduced at the furin cleavage site generates a novel N-glycosite at position N300. NanoLC-MS/MS results also showed that N276 was occupied by glycans, despite not being included in a canonical sequon (Supplementary Tables S2, S3).

**Figure 4:**
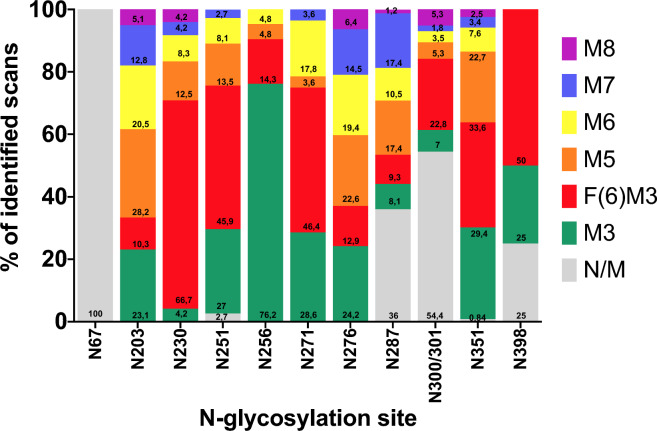
Estimation of *N*-glycan distribution in the different sBLV-EnvFm N-glycosylation sites. The relative abundance of *N*-glycans identified by nanoLC-MS/MS at each potential glycosite (calculated as the proportion of spectra hits resumed in Supplementary Table S2) are displayed as the relative proportion of modified (coloured) versus non-modified (grey) peptides. The plot summarizes all glycoforms found at each potential N-glycosylation site along with unmodified peptides. N129 was not detected in any spectra. Structure abbreviations: *F(6)M3* Man_3_GlcNAc_2_Fuc, *M3* Man_3_GlcNAc_2_, *M5* Man_2_Man_3_GlcNAc_2_, *M6* Man_3_Man_3_GlcNAc_2_, *M7* Man_4_Man_3_GlcNAc_2_, *M8* Man_5_Man_3_GlcNAc_2_, *N/M* non-modified (non-glycosylated).

Finally, the percentage of non-glycosylated (unmodified) vs N-glycosylated peptides per site, as well as the relative abundance of the different glycans in each N-glycosylated site, was estimated by spectral counting analysis using nanoLC-MS/MS (Fig. 4, Supplementary Tables S2 and S3). Peptides containing glycosites N203, N230, N256, N271 and N276 were only identified as glycosylated peptides, while the percentage of scans containing unmodified N251 and N351 was very low (2.7% and 0.84%, respectively), suggesting high glycan occupancy. The rest of glycosites (N287, N300 and N398) showed scans corresponding to unmodified peptides ranging between 25 and 54%.

### sBLV-EnvFm discriminates between positive and negative BLV samples showing high antigenicity

**Figure 5:**
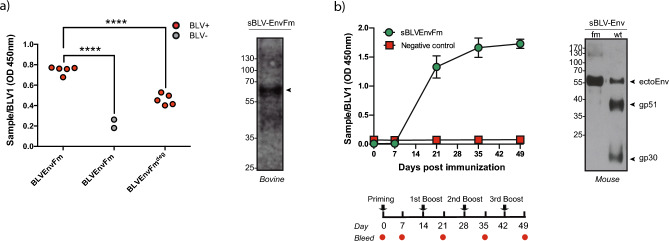
Antigenic and immunogenic properties of recombinant sBLV-EnvFm. (**a**, left) ELISA reactivity of BLV + (red filled dots) and BLV- (grey filled dots) bovine field sera against sBLV-EnvFm is shown. PNGase F-treated sBLV-EnvFm^deg^ was also tested by ELISA against BLV + sera showing a significant reduction in reactivity (****p < 0.0001, one-way ANOVA). The data are expressed as the mean ± 3 s.d. of the negative controls and were normalized against BLV1 positive control. (**a**, right): Western Blot analysis showing reactivity of field bovine BLV + serum against recombinant SEC-purified sBLV-EnvFm. A single band of > 55 kDa is visualized corresponding to sBLV-EnvFm protein (arrow). (**b**, left) ELISA showing kinetics of sBLVEnvFm-specific IgG titers during immunization of BALB/c mice (n = 4 per group). Mice were immunized subcutaneously at day 0 (priming) and at weeks 2, 4 and 6 (1st, 2nd and 3rd boosts represented with black arrows). Serum samples were collected at days 0, 7, 21, 35 and 49 post-immunization (indicated by red dots). The data obtained with 1:1000 dilutions of sera were normalized against a BLV1 positive control. The cutoff point was calculated as the average of true negative sera ± 2 standard deviations (0.042 ± 0.072). Sera obtained from pre-immunization mice were considered true negatives. Sera were considered positive if it had OD values above the cutoff (0.114). Filled green circle: sBLV-EnvFm immunization. Filled red squares: PBS (negative control) immunization. (**b**, right) Western Blot analysis showing reactivity of an anti-sBLV-EnvFm immune murine serum against recombinant SEC-purified sBLV-EnvFm (> 55 kDa single band) and affinity-purified sBLV-Env*wt* (> 55 kDa band corresponding to unprocessed sBLV-Env*wt*, > 35 kDa band corresponding to gp51 and a 20 kDa band corresponding to gp30 ectodomain). Blots were cropped to improve clarity, original blots are presented in Supplementary Fig. S11.

sBLV-EnvFm antigenicity was evaluated by ELISA with bovine sera previously tested for BLV using a commercial ELISA^51^. Recombinant sBLV-EnvFm showed a significant reactivity with BLV + sera, being able to discriminate between positive and negative field samples (p < 0.0001) (Supplementary Fig. S8). Concomitantly, glycan contribution to antibody recognition within natural infected animals was analysed by ELISA using a PNGase F treated antigen (sBLV-EnvFm^deg^) (Fig. 5a, left). Partial deglycosylation of sBLV-EnvFm generated a significant drop in reactivity with specific BLV + antibodies when compared to its glycosylated counterpart (sBLV-EnvFm, p < 0.0001). Positive BLV field bovine serum reactivity against sBLV-EnvFm protein was also analysed by western blot with a BLV + serum, which specifically recognizes sBLV-EnvFm showing the > 55 kDa protein band (Fig. 5a, right; Supplementary Fig. S9).

### sBLV-EnvFm demonstrates strong immunogenicity and specific antibody response in BALB/c mice

Next, sBLV-EnvFm immunogenicity was evaluated in BALB/c mice (Fig. 5b, left). sBLV-EnvFm specific antibodies were analysed by ELISA at days 0, 7, 21, 35 and 49 after immunization. An increase in specific antibody levels was detected after the first booster followed by a sustained response with successive doses demonstrating that sBLV-EnvFm is immunogenic in a murine model generating a strong specific humoral response**.**

In order to complete our comparative analysis between furin-mutated and *wild type* BLV-Env ectodomains, we measured western blot crossreactivity of sBLV-EnvFm and sBLV-Env*wt* proteins with sera from sBLV-EnvFm-immunized mice. As shown in Fig. 5b (right), sBLV-EnvFm was recognized as a single > 55 kDa protein band. Interestingly, reactivity with sBLV-Env*wt* protein revealed 3 bands corresponding to non-cleaved BLV-Env*wt* (> 55 kDa) ectodomain as well as gp51 (> 35 kDa) and gp30 ectodomain (20 kDa) as seen by SDS-PAGE in Supplementary Fig. S2.

## Discussion

The development of an effective BLV vaccine based on Env has been a difficult task^27,52–59^. Many groups have tried to express the gp51 subunit in various systems to enhance its expression for both vaccine and diagnostic purposes^40,55,60–64^. Therefore, a well-characterized BLV Env protein that is easy to produce is essential for antigen/immunogen design and structural studies.

In this work sBLV-Env proteins (sBLV-Env*wt* and sBLV-EnvFm) were successfully produced in *Drosophila* S2 cells with high protein yields (over 10 mg L^−1^).

sBLV-Env*wt* protein was efficiently expressed in S2 cells, but it tended to form aggregates of high molecular weight non-related to the expected oligomeric distribution. More importantly, gp51-gp30 complex showed high dissociation during the purification process with a loss of non-tagged gp51. While this phenomenon, triggered by furin-like protease processing, is expected in deltaretrovirus Env, it also signifies a loss of epitopes of utmost importance in our purification strategy.

To stabilize the gp51–gp30 complex we introduced a substitution in the furin cleavage site (furin mutated, Fm) generating a proteolytic processing defective mutant, to improve BLV Env purification. González-Reyes et al*.*^65^ employed a similar strategy by mutating the furin site of hRSV fusion glycoprotein, resulting in inhibition of syncytia formation and alterations in trimers’ shape. We think a similar scenario could occur with sBLV-Env Fm, with gp51-gp30 complexes retaining all antigenic epitopes and constraining the presence of different Env conformers that could divert the activation of the immune system towards the recognition of non-neutralizing epitopes***.*** In this regard our group has recently demonstrated that a similar BLV-EnvFm protein containing the transmembrane (TM) and cytosolic domains is able to form VLPs when co-expressed with Gag protein^42^. In the present work, this construct was modified by replacing the TM domain with a C-terminal Streptag to obtain a soluble protein easy to purify and quantify.

sBLV-EnvFm was purified by SEC showing several fractions with different oligomerization states. The monomeric fraction was analyzed by SEC-MALS showing high polydispersion evidenced by the presence of several populations with variable molecular weights. This can be explained by a potential dynamic equilibrium between dimer/trimer and monomer predominant forms. The presence of high molecular weight-oligomeric species can also be attributed to disulfide bond contribution as was demonstrated under non-reducing conditions where bands corresponding to high molecular weight species (130 kDa and > 170 kDa) were observed. As cysteine 212 involved in intersubunit disulfide isomerization remains reactive^66^ it can contribute to the formation of intra or intermonomeric disulfide bonds (C212 oxidation in nanoLC MS/MS results showed it is reactive). For human deltaretrovirus HTLV it was also demonstrated that high-sized oligomers could be related to potential disulfide-linked intra and intersubunit species^67^.

Glycosylation plays a critical role in viral life cycle (mainly in the early steps of viral infection)^68^. It promotes the proper folding and subsequent trafficking, affecting protein stability, conformation and consequently its antigenicity. Thus modification of one or several glycosylation sites can dramatically impact virus survival and transmission^33^. As viral glycosylation is undergone using host’s cellular machinery, viral proteins contain mostly host-derived self-like glycans. As these molecules are broadly non immunogenic, viruses employ glycans as a shield to avoid recognition of antibody-sensitive viral protein determinants, thus evading immune recognition^69^. In HIV-1, this glycan “mask” reduces antigenicity of Env as *N-*glycans protect the virus from host neutralizing antibodies during infection^36^. However, it has been demonstrated that a fraction of *N-*glycans is readily recognized by broad neutralizing antibodies^70,71^ making them significant antigenic determinants. This was demonstrated for HIV-1, where certain broad neutralizing antibodies can exclusively recognize glycans within highly conserved mannose-rich epitopes of Env^72,73^.

In this work we characterized the *N-*glycan components of our recombinant sBLV-EnvFm protein. The predominant *N-*glycan structures observed across the protein ectodomain either by *N-*glycan analysis (NP-HPLC) or glycopeptide analysis (nanoLC/MS), demonstrates that proteins expressed in S2 cells present simple pauci/oligo mannosidic glycans, as previously reported, with a high component of (α-1,6) fucosylated glycans^74,75^. Assignation of all predicted N-glycosylation sites (N129, N203, N230, N251, N256, N271, N287, N351, N398) was performed in our recombinant protein in accordance with previously published studies^23,37–39^.

N67 site was not glycosylated as previously reported by Rizzo et al. and others^37,39–41,76^. Mutations at the furin cleavage site created a N-glycosylation site at N300 that was unexpectedly glycosylated. NanoLC-MS/MS analysis revealed that approximately 46% of the identified spectra contained N-glycosylated N300. In addition N276 (where a non-consensus sequon was present) was glycosylated as well. The introduction of these unprecedented potential N-glycosylation sites do not compromised protein expression and/or conformational epitopes recognized by conformational BLV1 mAb. The extent to which these newly introduced N-glycosylation sites impact immunogenicity needs to be addressed.

Proper recognition of conformational epitopes F, H and G of gp51 relies on glycosylation of N129^39^. Our results showed that anti-BLV sera were able to interact with our recombinant sBLV-EnvFm revealing the correct folding of the protein and the presence of a glycosylated N129. Our results are in accordance with other studies which suggested that N129 contains non-complex glycans similar to insect cell *N-*glycans composition that are recognized by conformational BLV1 mAb^25,39,41,42,77^. Unexpectedly, N129 glycopeptide could only be detected by MALDI-TOF MS analysis after PNGase F treatment and neither glycan occupancy nor the *N-*glycans attached to this N-glycosylation site could be assigned. We hypothesize that detection of the peptide containing N129 is probably masked by other modification or the presence of highly hydrophilic glycans counteracts direct glycopeptide detection by nanoLC MS/MS.

For most confirmed N-glycosylation sites, a high percentage of glycosylated N-linked glycan sequons of sBLV-EnvFm was observed except for N287 and N398, which presented a marked percentage of unmodified peptides (36 and 25%, respectively) in the total peptide pool.

While N203, N230 and N256, N271 and N276 appear to be 100% glycosylated, the remaining N-glycosylation sites showed from partial to high glycosylation occupancy levels. It seems that these N-glycosylation sites are super accessible sites for the N-glycosylation machinery and can accommodate glycans without affecting protein conformation and/or functionality. In addition it was previously reported that single-mutated N129, N203, N230 and N251 do not inhibit in vitro cell fusion nor in vivo infectivity^37,38,78^. The glycopeptide containing N203 has been previously identified by Olivero et al. in BLV VLPs meaning that this is one of the most abundant/accessible peptides present in recombinant sBLV-EnvFm expressed in Drosophila S2 cells as well^42^. Interestingly, N203 is located close to the CXXC motif involved in the gp51–gp30 covalent bonding^13^ and has been demonstrated to be essential for BLV in vitro infection^39^. Unfortunately, the lack of three-dimensional structural data of BLV Env trimer (or any other deltaretroviral Env proteins) poses challenges not only for structural modification design and engineering, such as disulfide bond stabilization or proline substitutions, as seen in HIV-1^79^ but also for *N-*glycan mapping, to understand the potential impact of glycans on protein function and immune recognition.

BLV serological diagnostic tests are principally based on gp51 antigen traditionally produced in an ovine BLV persistently infected cell line, FLK^80,81^. Production of high amounts of FLK-gp51 antigen requires partial purification and concentration and is time-consuming, expensive and laborious^82^.

We set up a proof of concept ELISA to test sBLV-EnvFm antigenicity. Our results showed that this protein was able to discriminate between positive and negative bovine field sera, being well recognized by anti-BLV natural antibodies despite the high occurrence of fucosylated oligomannosidic *N-*glycans. We observed a significant reduction in reactivity upon deglycosylation of the antigen, supporting the hypothesis that glycans are an important structural component, maintaining protein integrity as discussed before. In this regard, *N-*glycan loss (with the concomitant addition of a negative charge) might provoke an alteration in the protein structure by removing regions previously exposed to antibodies or by affecting the coating of ELISA plates. However, the existence of antibodies in the immune repertoire which directly recognize *N-*glycans as in HIV-1^83^ should not be excluded.

sBLV-EnvFm protein was also recognized by bovine sera as shown by WB, strengthening the ELISA results (using murine sera). A recombinant BLV gp51 expressed in TBSV showed similar reactivity using positive bovine sera when analysed by WB^63^. Thus, this recombinant protein expressed in S2 cells represents an excellent tool to detect specific anti-BLV antibodies. As gp51 can be altered by nucleotide and amino acid substitutions that may be located in epitopes related to neutralization^84^, the fact of including the entire ectodomain allows a better interaction with antibodies present in bovine sera. In this regard, it was recently demonstrated that naturally occurring H/G epitope-mutated gp51, expressed in baculovirus were less reactive to BLV positive cattle sera with conserved F, G and H epitopes^60^. The advantage of employing our sBLV-EnvFm protein as antigen would probably avoid drawback of BLV diagnosis giving a broad sera recognition. Enzootic bovine leukosis (EBL) poses a significant animal health problem that severely impacts dairy cattle, affecting production rates and imposing restrictions on live animal export. The lack of an effective vaccine and/or specific treatments against this viral disease necessitates the implementation of control strategies that can reduce viral transmission and lower the prevalence of infection. As previously mentioned, herd management based on “test and segregate” strategies, which involve eliminating animals with high proviral loads and lymphocytosis, currently represents the most effective method of reducing transmissibility with a tangible impact on herds. To achieve this goal, it is essential to perform accurate diagnoses using reliable tools. In this regard, the ELISA results obtained with sBLV-EnvFm show promising potential for future EBL control programs based on “test and segregate” strategies to further reduce BLV transmission.

Extracellular localization of gp51 in BLV Env conformation (when compared to gp30) renders this subunit completely exposed to the immune system being the most immunogenic^85^. Notably, recent studies showed that while gp51 stimulates the humoral immune response with massive expression of specific antibodies in infected animals, CD8^+^ T cells are better activated by gp30 portion as gp30 CTL epitopes appear to be less polymorphic, showing the relevance of producing an immunogen containing the complete ectodomain of BLV Env protein^86,87^. Recombinant sBLV-EnvFm resulted in a highly immunogenic protein with specific antibodies detected in high levels after 21 days after immunization/booster. This enhanced humoral immune response can be explained in part by the presence of α-1,6 fucose *N-*glycans, as these glycans can contribute to the development of a strong humoral immune response in mice^88,89^. In agreement with other studies, since this protein was expressed in insect cells with abundance of glycan motifs not present in mammalian cells, it can be recognized by bovine antibody repertoire making them highly immunogenic^90^. We think that the success of sBLV-EnvFm as potential immunogen can be associated to this recognition. We cannot discard the possibility that in the antibody repertoire of BLV infected animals some of them could recognize glycan motifs similar to bNAbs raised against mannose-rich epitopes in HIV-1 gp120^72,73^. Even if highly immunogenic, the neutralizing capacity of antibodies elicited by these immunogens must be addressed to definitely consider the possibility of employing sBLV-EnvFm proteins expressed in S2 cells for BLV immunogen development. In this regard the contribution of cell-mediated immunity against BLV, induced by sBLV-EnvFm should be evaluated.

Antibodies raised against sBLV-EnvFm were able to recognize not only sBLV-EnvFm but also recombinant sBLV-Env wild type (non-cleaved Env, gp51 and gp30). Thus, our immune polyclonal serum could be further employed to specifically detect gp30 since all commercial monoclonal antibodies can only recognize gp51 thus generating a high valuable reactive for fusion subunit discrimination.

In conclusion, we have successfully developed and characterized a recombinant soluble BLV Env furin mutated ectodomain protein capable of generating humoral immune response in mice and with a high antigenic potential for diagnosis. The furin mutation, along with the simultaneous introduction of a newly N-glycosylation site at N300, does not compromise its expression, secretion or the recognition by bovine sera. This indicates that our recombinant protein retains its antigenicity and immunogenic capacity. Furthermore, our production system guarantees cost-effective yields and remarkable reproducibility, making it ideal for meeting BLV field detection needs. The use of Drosophila S2 stable cell lines, adapted to grow in a protein-free medium, is a key step in avoiding contamination with cellular proteins or bovine seroalbumin present in fetal calf serum formulations. This is crucial for a potential vaccine approach. Our protein is highly antigenic and, despite being oligomannosidic, these insect *N-*glycans enable the correct binding of antibodies to the recombinant protein, making it a valuable tool for diagnosis and an excellent candidate for a vaccine strategy against BLV infection.

## Methods

### Plasmids and constructs generation for protein expression in Drosophila S2 cells

Partial DNA sequence (1215 bp) of the *BLV-env* gene (GenBank access number EF600696, nucleotides 4826–6371) lacking the coding sequences of the N-terminal signal peptide (nucleotides 4826–4921) and the C-terminal amino acids of gp30 (coding the membrane spanning and cytosolic domains, nucleotides 6140–6371) was codon-optimized and synthetized (Genscript) for efficient expression in Drosophila Schneider’s 2 cells^91^ (Thermo Fisher Scientific). Codon-optimized BLV *env* gene was further cloned in expression plasmid pT350 (a modified pMT/BiP vector^47^) in order to express the soluble ectodomain (aa 33–437) of BLV Env protein (sBLV-Env*wt*) (Fig. 1a).

This construct was further modified in order to obtain a mutant form lacking the natural furin cleavage site (RVRRSPV) to avoid proteolytic processing to confer stabilization (sBLV-EnvFm, Fig. 1a). Site-directed mutagenesis was assessed by modifying the double basic sequence RR between gp51 and gp30, by introducing substitutions R300N and R301N precluding furin cleavage site^65,92,93^. To ablate furin cleavage site, a double mutant (R300N/R301N) (sBLV-EnvFm) was designed (Fig. 1a).

The expression plasmid contains the described fragments preceded by an N-terminal *Drosophila* BiP secretion signal which drives efficient translocation of the nascent protein into the ER of the transfected cell, and its expression is under control of *Drosophila* metallothionein promoter (pMT) inducible by CdCl_2_. At the C-terminal end of the constructions, the plasmid contains a tandem *Strep-tag II* sequence to allow efficient purification by affinity chromatography^47^.

### Generation of inducible stable Drosophila S2 cell lines producing soluble BLV-Env proteins

sBLV-Env*wt* and sBLV-EnvFm proteins were obtained from stable Drosophila S2 cell lines as described below.

Twenty-four hours before transfection, S2 cells were seeded in T25 flasks (1 × 10^6^ cells/mL) and grown at 28 °C in Schneider’s Drosophila medium supplemented with 10% heat-inactivated fetal bovine serum (GIBCO), 50 U/mL penicillin and 50 µg/mL streptomycin (penicillin/streptomycin, GIBCO). Drosophila S2 cells were co-transfected with 2 µg of plasmid constructs (pMT/BiP/BLV-Env*wt* or pMT/BiP/BLV-EnvFm) and a plasmid encoding puromycin acetyltransferase (0.1 µg)^94^ as dominant selectable marker, using Effectene Transfection Reagent (QIAGEN) according to the manufacturer’s recommendations. Stable cell lines expressing BLV Env ectodomain proteins were obtained by antibiotic selection using 6 µg/mL puromycin (InvivoGen). After selection, cells were adapted stepwise to a protein-free growth medium (Insect-XPRESS, Lonza) for 4 weeks to generate two stable cell lines BLV-Env*wt*-S2 and BLV-EnvFm-S2 expressing sBLV-Env*wt* and sBLV-EnvFm proteins respectively upon induction with 5 µM CdCl_2_ (Sigma-Aldrich).

### Expression and purification of recombinant BLV envelope glycoprotein

Serum-free adapted cells BLV-Env*wt*-S2 and BLV-EnvFm-S2 were cultured with agitation at 28 °C to a final density of 7 × 10^6^ cells/mL in Erlenmeyer flasks, and induced with 5 µM CdCl_2_ (Sigma-Aldrich) during 30 h. Cells were removed by centrifugation at 6000×*g* for 30 min at 4 °C and supernatant containing secreted proteins was further centrifuged and filtered through 0.22 µm. Purification was performed by affinity chromatography using pre-packed Strep Trap HP columns (GE Healthcare) followed by size exclusion chromatography (SEC) using a Superdex 200 16/60 column (GE Healthcare) in 10 mM Tris (pH 8), 150 mM NaCl. Pure protein (sBLV-Env*wt* and sBLV-EnvFm) was analyzed by SDS-PAGE, confirmed by mass spectrometry, quantified spectrophotometrically and concentrated before proceeding. Precision Plus Protein Dual Color Standard (15–250 kDa Biorad) was used as a molecular weight ladder. Combined MALDI-TOF and nanoLC-MS/MS analysis (see “Methods” below) allowed the identification of sBLV-EnvFm achieving more than 90% of sequence coverage (Supplementary Fig. S7).

The molecular weight and the oligomerization state of soluble recombinant BLV Env proteins were determined using multi angle laser light scattering coupled in line to size exclusion chromatography (SEC-MALS). Superdex 200 10/300 (GE Healthcare) column equilibrated with 10 mM Tris (pH 8.0), 150 mM NaCl was connected to a MALS instrument (DAWN HELEOS II, Wyatt Technologies, Santa Barbara, CA, USA). Differential refractive index was performed in line using an Optilab T-rEX detector (Wyatt Technology). Data was analyzed with ASTRA software (Wyatt Technology) in order to calculate the average molar mass of the multimeric species as well as its mass distribution.

### Dynamic light scattering

sBLV-EnvFm was analyzed by DLS in a Zetasizer Nano S (Malvern). Measurements were done in triplicates at 4 °C using disposable plastic cuvettes (UVette, Eppendorf) and Hydrodynamic Radius (RH) for each population is reported as the averaged media of each peak (± standard distribution).

### Western blot

Protein concentration from purified sBLV-EnvFm and sBLV-Env*wt* was quantified using adsorption at UV280nm and with Pierce™ BCA Protein Assay Kit (23227, Thermo Scientific). Eight micrograms of protein were loaded under reducing or non-reducing 12% SDS-polyacrylamide gels. After electrophoresis, samples were electroblotted onto a nitrocellulose membrane for 90 min at 300 mA and 4 °C (Hybond-ECL, Cytiva). Membranes were blocked overnight at 4 °C 3%(w/v) BSA in PBS. Primary monoclonal antibody mouse anti-gp51 to the D–D′ epitope BLV2: 1/2000 (VMRD Inc., Pullman, WA, USA) was incubated for 1 h at room temperature. Membranes were vigourously washed with PBS + 0.3%(v/v) Tween 20 and incubated for 1 h at room temperature with goat anti-mouse monoclonal antibody conjugated to horseradish peroxidase-HRP (1/5000, Santa Cruz Biotechnology). Finally, the assay was developed with SuperSignal™ West Pico Chemiluminescent Substrate (Thermo Fisher Scientific), exposed to Hyperfilm ECL (Cytiva) and manually developed. Page-Ruler Prestained Protein Ladder, 10–180 kDa (Thermo Fisher Scientific) was used as a molecular weight ladder.

For western blot analysis using mouse serum (1/500 dilution), sBLV-EnvFm and sBLV-Env*wt* were loaded in equal amounts. sBLV-EnvFm was also employed as antigen to test BLV + bovine serum reactivity (dilution1/500) using a goat anti-bovine IgG HRP as secondary antibody. In both the procedure described above (incubation times, wash steps and development) was the same.

### Enzyme-linked immunosorbent assay

Briefly, 96-wells plates (Nunc Maxisorp) were coated overnight at 4 °C with 0.5 µg of purified sBLV-EnvFm diluted in 50 mM carbonate buffer (pH 9,6). Protein-coated wells were saturated with 3%(w/v) non-fat dry milk + 0,2% (v/v) Tween 20 in PBS for 1 h at 37 °C. Field BLV sera samples were diluted 1/500 in PBS-5% non-fat dry milk-0.2% Tween 20 and incubated for 1 h at 37 °C. These sera samples (both positive or negative for BLV infection) were previously screened using a commercially available Bovine Leukemia Virus Antibody Test Kit (VMRD Inc., Pullman, WA, USA) which detects antibodies against BLV gp51 protein in bovine serum^51^. Samples were further confirmed by *env* gp51 PCR amplification as described in^95^. Monoclonal antibody against a conformational epitope on gp51 (1/1000 dilution) was used as a positive control (BLV1, VMRD Inc., Pullman, WA, USA). Sera from mice immunized with sBLV-EnvFm was included (see below in Immunization studies). Wells were washed five times with PBS- 0,1% Tween 20 and then incubated with rabbit anti-bovine IgG-HRP antibody (Sigma-Aldrich) or goat anti-mouse IgG-HRP antibody (Santa Cruz Biotechnology) diluted 1:10,000 and 1:5000 respectively, in PBS-5% non-fat dry milk- 0,2% Tween 20 for 45 min at room temperature. After 5 washes with PBS-0.1% Tween 20 the assay was revealed with 50 µL per well of TMB substrate (50 µg/mL, Sigma-Aldrich) and H_2_O_2_ (0.003%) in 0.11 M acetate buffer pH 5.5. Reaction was stopped with 3N H_2_SO_4_ and absorbance was measured at 450 nm (Multiskan FC Microplate Reader, Thermo Scientific).

For glycosylation studies 100 µg of sBLV-EnvFm were deglycosylated in non-denaturing conditions with PNGase F (NEB) following manufacturer’s instructions and assayed in an ELISA assay as describe above.

### Glycan purification by NP-HPLC and exoglycosydase treatment

BLV Env glycosylation sites prediction was assessed using NetNGlyc server 1.0 software^96^. Canonical N-glycosylation sites have the sequon Asn-X-Ser/Thr (X being any amino acid except proline).

Predictions above a threshold of 0.4 (the program fixed par default 0.5) were considered as a potential N-glycosylation site. This criterion was based on previous studies where N256 and N351 were assigned as N-glycosylation sites^39,76^.

PNGase F-glycan release followed by 2-aminobenzamide (2AB) labelling and separation of the 2AB derivatives on normal phase HPLC was performed to analyze composition of purified *N-*glycans in sBLV-EnvFm as follows. One hundred micrograms of sBLV-EnvFm were denatured in a solution containing 5% SDS in 50 mM buffer phosphate for 10 min at 95–100 °C and then cooled to RT. 1% NP40 final concentration was added and *N-*glycans were removed by treatment with 500U PNGase F (NEB) in buffer phosphate pH 7.6 overnight at 37 °C with agitation. *N-*glycans were collected and further purified by ethanolic precipitation (70%). Non-derivatized oligosaccharides were then purified in graphite column (Glycoclean H, Prozyme, Cat. GKI 4025) and finally eluted in 50% v/v acetonitrile/0.1% trifluoracetic acid. Each fraction was eluted separately. Samples were dried in a centrifugal vacuum evaporator without heating for 2AB labeling^97^. *N-*glycans as 2AB derivatives were analyzed by high performance liquid chromatography (HPLC) using a normal phase (NP) column^98^. NP-HPLC was conducted using a TSK-GEL Amida-80 column (250 94.6 mm TosoHaas, Japan) with 50 mM formiate pH 4,4 (as solvent A) and acetonitrile as solvent B at a flow rate of 0.4–1 mL/min. Structural assignment was performed by comparison of experimental glucose units values (GU) calculated for each fraction on the chromatogram and compared to theoretical GU values reported in the Glycostore database (http://www.glycostore.org) for the assignment of possible structures linked to the sBLV-EnvFm glycoprotein. Migration times of the samples were normalized by standards (dextran). *N-*glycan relative proportion (%) was calculated assuming that there was a direct (linear) proporcionality between fluorescence intensity (peak) and the glycan mass and also assuming that all glycan were derivatized in the same proportion in the HPLC spectra. *N-*glycan relative abundance was calculated as a percentage considering the total area under the HPLC curve (GU) and for each peak each area was calculated considering 100% the integration of all areas of the curve. Retention times (min) of each fraction were expressed in glucose units (GU) calculated from a dextran hydrolysate analyzed under the same HPLC separation conditions.

To confirm *N-*glycan structures, 2AB derivatized *N-*glycans (1 nmol) coming from recombinant sBLV-EnvFm were treated with different exoglycosydases in a sequential digestion and further analyzed by HPLC.

The following enzymes (from Sigma-Aldrich) were employed in the digestion:

α2-3,6,8,9-Arthrobacter ureafaciens sialidase (ABS), Bovine kidney α l-fucosidase (BKF), Bovine testes β-galactosidase (BTG), *Streptococcus pneumoniae* β-hexosaminidase (GUH, SPH), Jack bean α-mannosidase (JBM). Digestion was done in a sequential manner in the same order as written above. Labeled *N-*glycans were digested in 50 mM sodium acetate buffer for 18 h at 37 °C. Enzymes were then removed by filtration with Amicon Micropure-EZ.

### Mass spectrometry analyses

Sample preparation for MS analyses was achieved with soluble proteins or bands from Coomassie-stained SDS-PAGE as described before^46^.

#### MALDI-TOF/TOF

For peptide mass fingerprinting, digestion was carried out by in-gel trypsin treatment (Sequencing-grade Promega) overnight at 37 °C. Peptides were extracted from the gels using 60% acetonitrile in 0.2% TFA, concentrated by vacuum drying and desalted using C18 reverse phase micro-columns (OMIX Pippete tips, Varian). Peptide elution from micro-column was performed directly into the mass spectrometer sample plate with 3μL of matrix solution (α-cyano-4-hydroxycinnamic acid in 60% aqueous acetonitrile containing 0.2% TFA). Mass spectra of digestion mixtures were acquired using a 4800 MALDI TOF-TOF Analyser (Applied Biosystems) in reflector mode and were externally calibrated using a mixture of peptide standards (Applied Biosystems). Collision-induced dissociation MS/MS experiments of selected peptides were performed.

Glycan analysis by MALDI-TOF was performed as previously described^99^. Briefly, 2-AB derivatized *N-*glycans were dissolved in water and 1 μL was mixed with 1 μL of a saturated solution of 2,5-dihydroxybenzoic acid in acetonitrile (Sigma-Aldrich) on the MALDI plate. The co-crystal was washed with 1 μL of absolute ethanol (Sigma-Aldrich). The positive reflector mode mass spectra were acquired on a 4800 MALDI-TOF/TOF (AB Sciex, Framingham, MA). Theoretical m/z were calculated using Glycan Mass Calculator (https://glycomass.com/glycan_calculate) according to : glycan composition, 2-AB labeling, sodium adduct [M + Na]+.

#### LC-MS/MS

Tryptic peptides were analyzed on a nanoLC system (UltiMate 3000, Thermo Fisher Scientific) coupled to a Q-orbitrap mass spectrometer (Q-Exactive Plus, Thermo Scientific). Peptides were separated on a 75 μm × 50 cm, PepMap RSLC C18 column, and eluted using a 90 min linear gradient from 5 to 40% acetonitrile in 0.1% formic acid, at a constant flow rate of 250 nL/min. The mass spectrometer was operated in a data -dependent acquisition mode using a top 12 method.

Protein and peptide identification was performed with PatternLab for Proteomics V (10.1038/s41596-022-00690-x) using a target-decoy database containing *Drosophila melanogaster* sequences downloaded from Uniprot (June 2020) to which the sequences of recombinant of bovine Leukemia Virus sBLV-EnvFm protein and usual proteomics contaminants was added. Searching parameters were set as follows: Peptide mass tolerance 35 ppm, variable modifications on N: Hex3HexNAc2 (+ 892.3173); dHex Hex(3) HexNAc(2) (+ 1038.3751); Hex(5)HexNAc(2) (+ 1216.4229); Hex(6)HexNAc(2) (+ 1378.4757); Hex(7)HexNAc(2) (+ 1540.5285) and Hex(8)HexNAc(2) (+ 1702.5813). For specific searches the presence of disulfide bonds was also considered. Peptide spectrum matches were filtered using a 10-ppm tolerance and FDR ≤ 1% at the protein level.

The mass spectrometry proteomics raw data have been deposited to the ProteomeXchange Consortium via the PRIDE (The PRIDE database resources in 2022: A Hub for mass spectrometry-based proteomics evidences. Nucleic Acids Res 50(D1):D543-D552 (PubMed ID: 34723319) partner repository with the dataset identifier PXD049277.

### Immunization studies

Specific-pathogen-free (SPF) 6–8 weeks old female BALB/c mice were housed at the Laboratory Animals Biotechnology Unit (Institut Pasteur de Montevideo). Groups of 4 animals were immunized subcutaneously with sBLV-EnvFm protein at days 0, 14, 28 and 42. sBLV-EnvFm protein was formulated with Freund’s adjuvant at 10 µg, using PBS formulated in Freund’s adjuvant as a negative control. Blood samples were obtained at days 0, 7, 21, 35 and 49 from submandibular vein for detecting humoral response. Serum samples were collected by centrifugation and kept at − 20 °C until use. sBLV-EnvFm specific antibodies were analysed at days 0, 7, 21, 35 and 49 after immunization by ELISA. Mice were deeply anesthetized with a mixture of xilazine/ketamine and cervical dislocation was performed by an experienced technician after final bleeding.

Emulsion (protein + adjuvant) was prepared in 1 mL (mixed in a 1:1 ratio vol:vol). Fifty microliters were injected subcutaneously in 4 different sites of the animal (total volume 200 µL). Complete Freund’s adjuvant (Sigma-Aldrich) was employed for the first immunization and incomplete Freund’s adjuvant for the boosters.

BALB/c mice were immunized by the Laboratory Animals Biotechnology Unit (Institut Pasteur de Montevideo). The experimental protocol was approved by the institutional "Ethics committee on animal experimentation" from Institut Pasteur de Montevideo, according to national law #18.611 and relevant international laboratory animal welfare guidelines and regulations. The Commission understands that the above mentioned protocol meets with all applicable legal standards in research (Law 18,611). All procedures were approved by the institutional ethics committee and we confirm that the study was reported in accordance with the ARRIVE guidelines.

### Statistical analysis

Statistical analysis was performed using GraphPad Prism6 (GraphPad Software, Inc., La Jolla, CA, USA). Statistical tests were calculated as ordinary one-way ANOVA with ****p < 0.0001.

### Supplementary Information

Supplementary Figure S1.

Supplementary Figure S2.

Supplementary Figure S3.

Supplementary Figure S4.

Supplementary Figure S5.

Supplementary Figure S6.

Supplementary Figure S7.

Supplementary Figure S8.

Supplementary Figure S9.

Supplementary Figure S10.

Supplementary Figure S11.

Supplementary Legends.

Supplementary Table S1.

Supplementary Table S2.

Supplementary Table S3.

Supplementary Table S4.

## Data Availability

The datasets generated during the current study are available from the corresponding author on reasonable request. All data generated in this study are included in this published article.
