# Supplementary figures and images for: Characterization and application of recombinant Bovine Leukemia Virus Env protein
Source: Sci Rep. 2024 May 28;14:12190. doi: 10.1038/s41598-024-62811-8 (PMC11133380; doi:10.1038/s41598-024-62811-8)

Figure S1

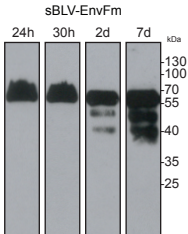

Supplement: Supplementary file 1 — Supplementary Figure S1. [file 41598_2024_62811_MOESM1_ESM.pdf]

Figure S2

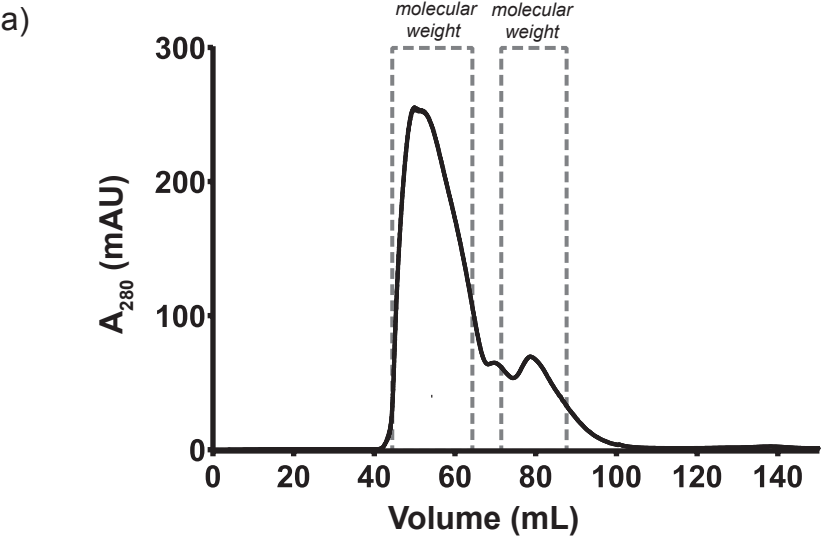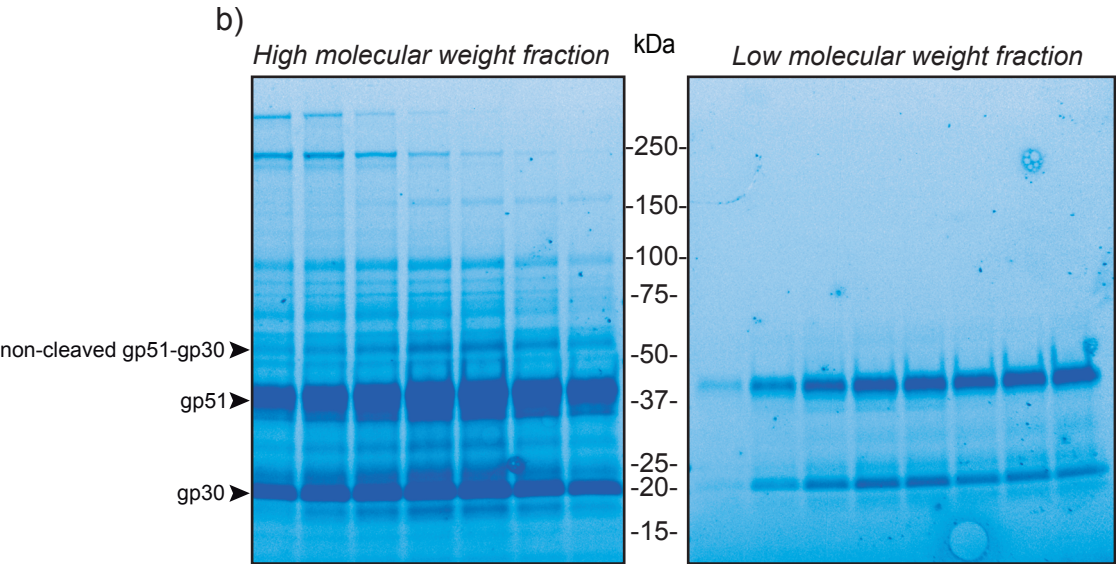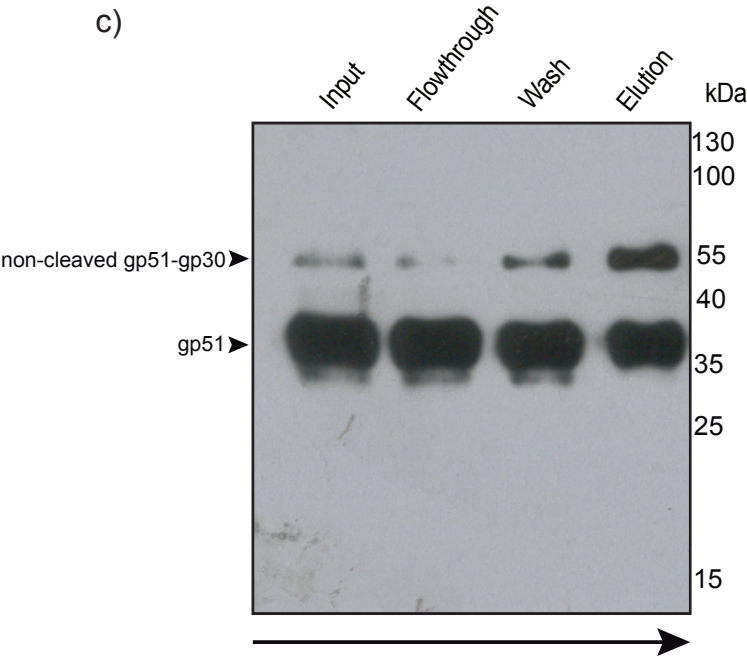

Supplement: Supplementary file 2 — Supplementary Figure S2. [file 41598_2024_62811_MOESM2_ESM.pdf]

**Figure S3**

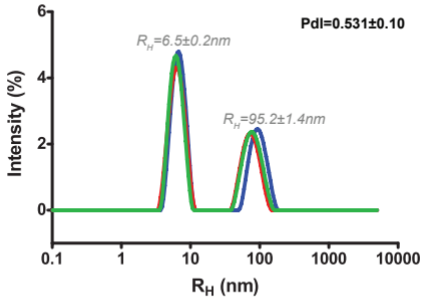

Supplement: Supplementary file 3 — Supplementary Figure S3. [file 41598_2024_62811_MOESM3_ESM.pdf]

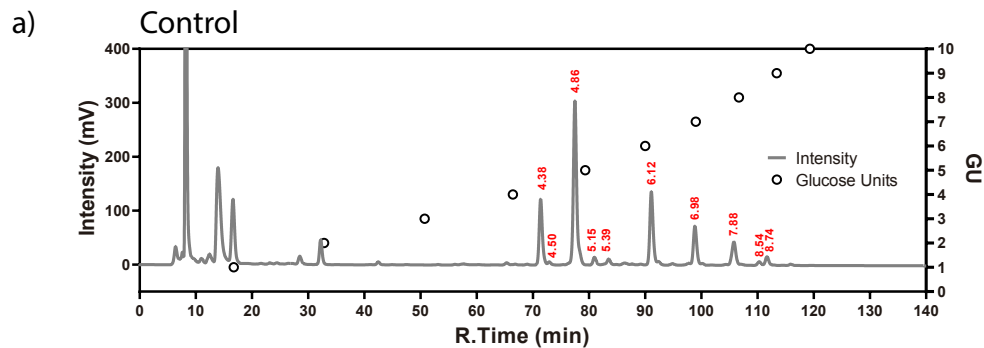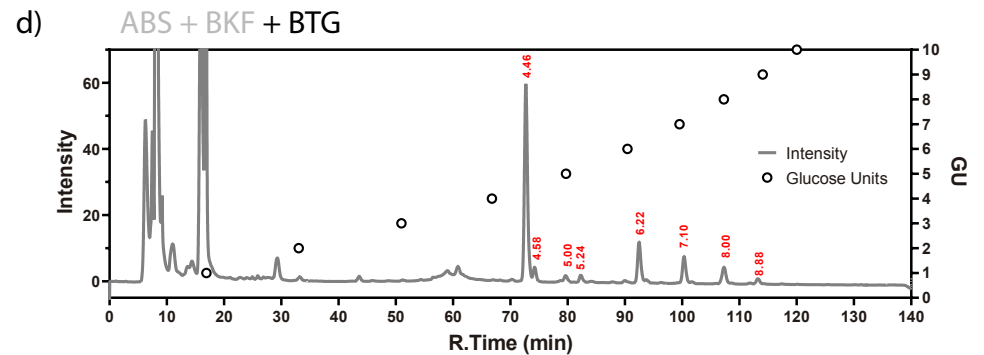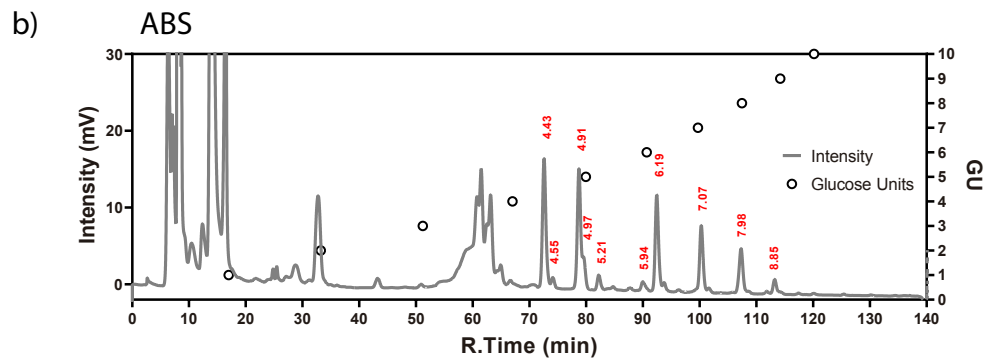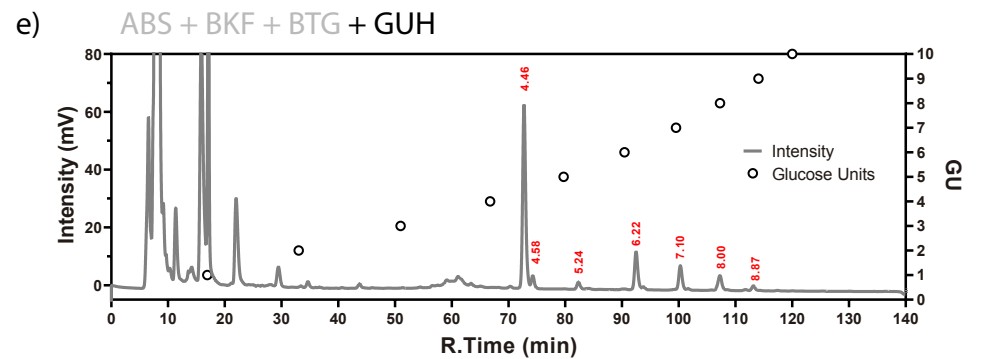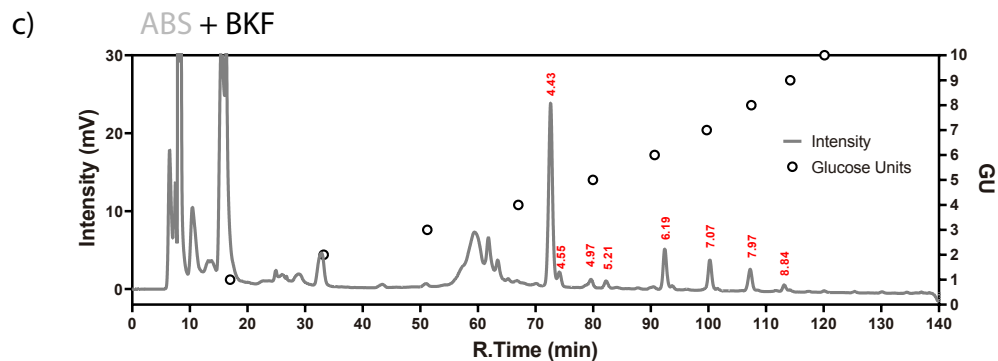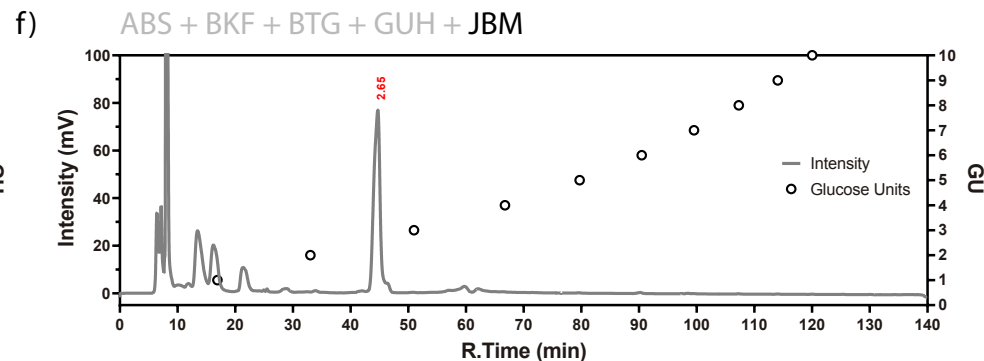

Supplement: Supplementary file 4 — Supplementary Figure S4. [file 41598_2024_62811_MOESM4_ESM.pdf]

Figure S5

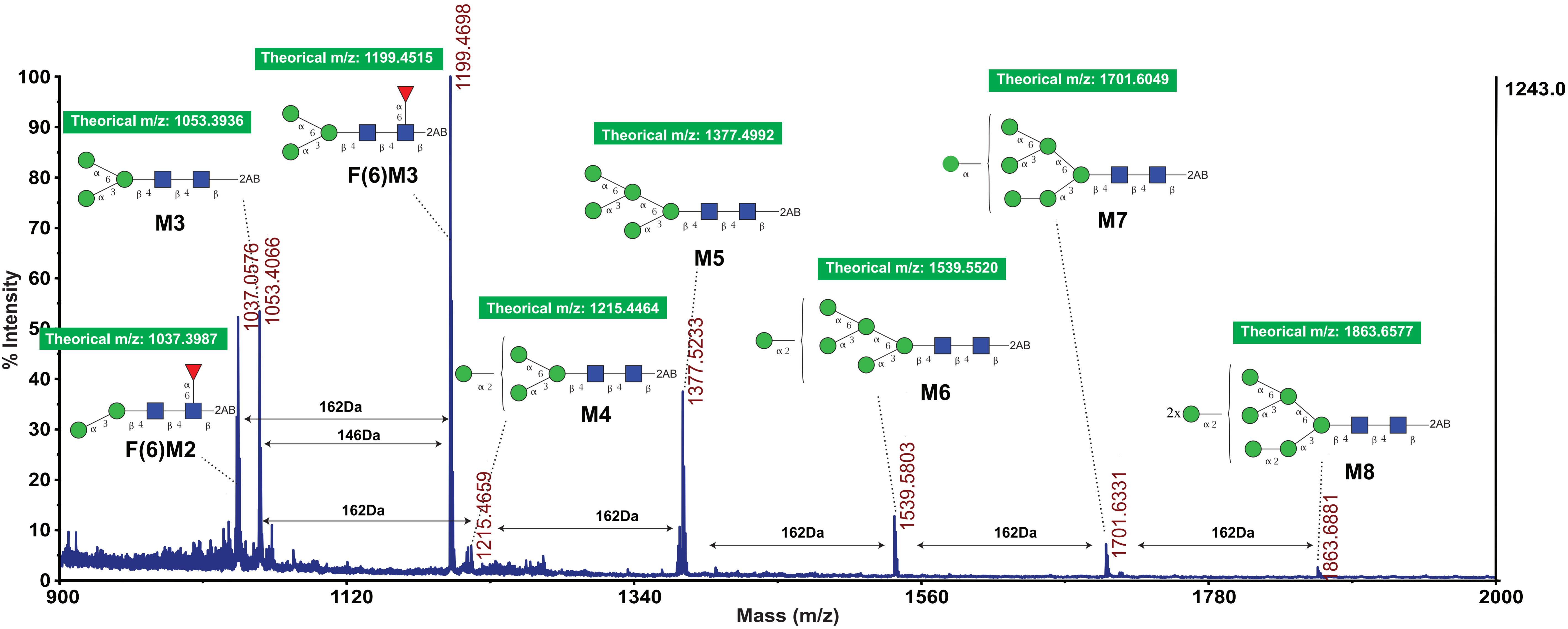

Supplement: Supplementary file 5 — Supplementary Figure S5. [file 41598_2024_62811_MOESM5_ESM.pdf]

Figure S6

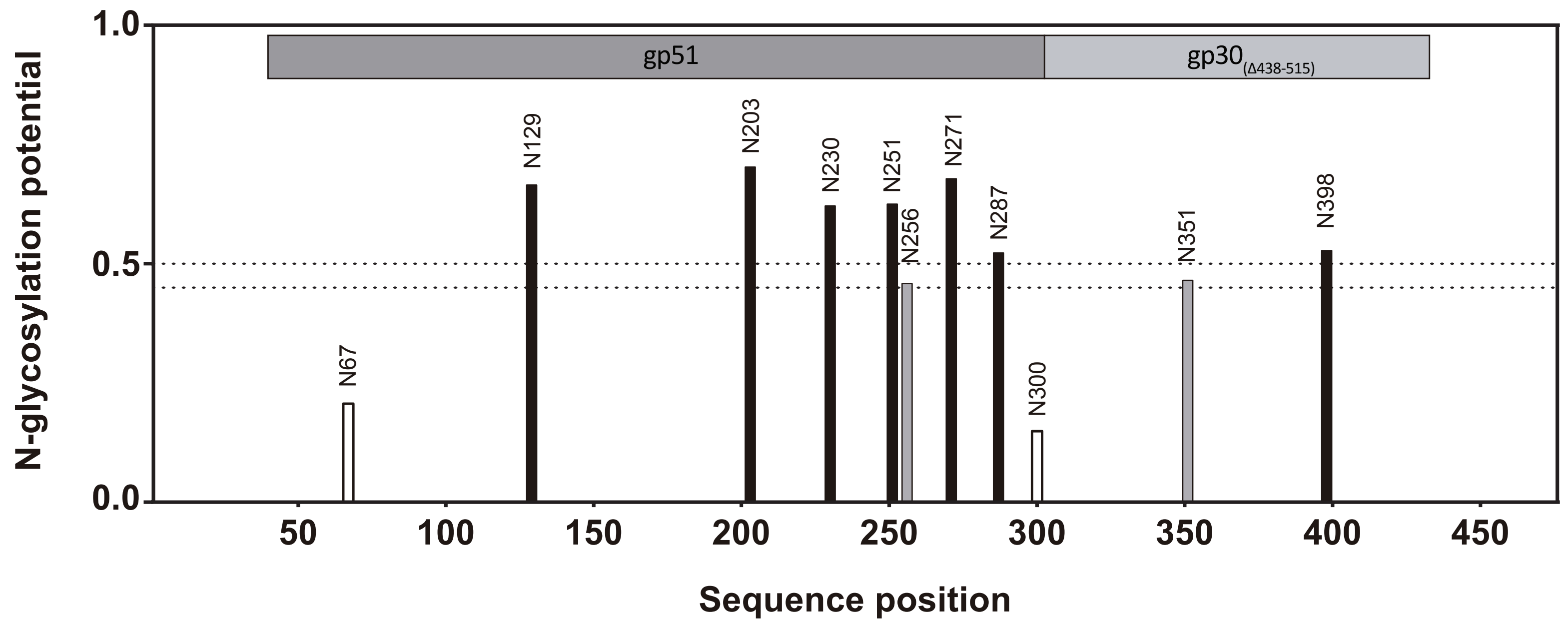

Supplement: Supplementary file 6 — Supplementary Figure S6. [file 41598_2024_62811_MOESM6_ESM.pdf]

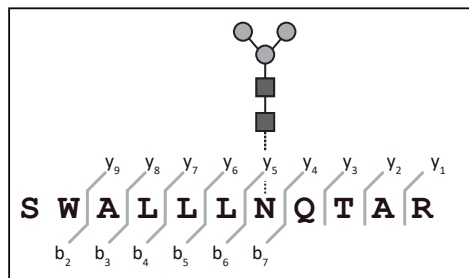

SCAN 17779

Z=+2

measured m/z = 1083.017

Theoretical m/z = 1083.0152

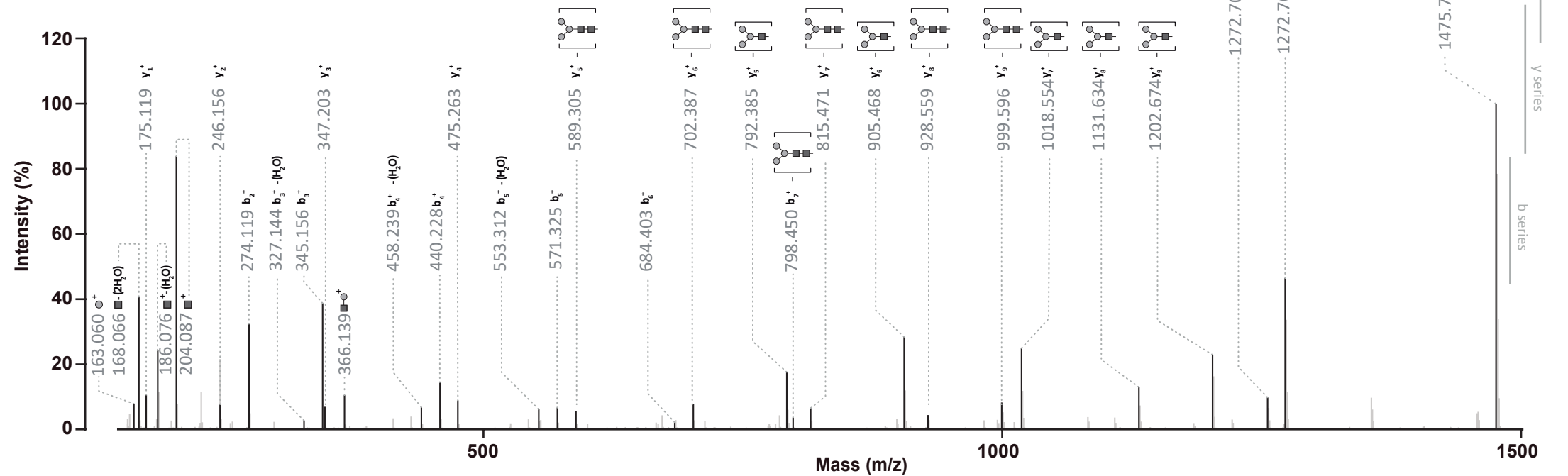

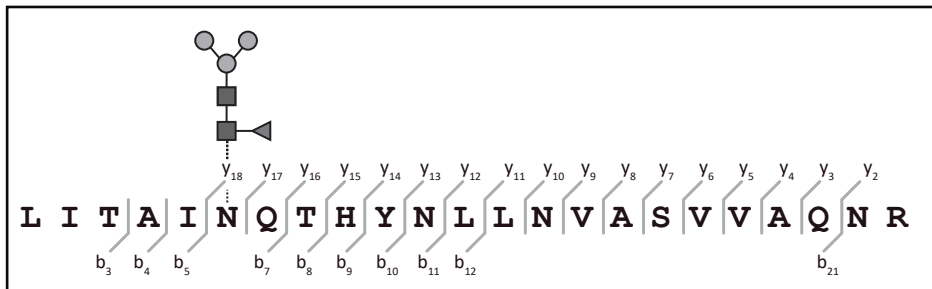

SCAN 17658  
Z=+2  
measured m/z = 1795.404  
Theoretical m/z= 1795.8884

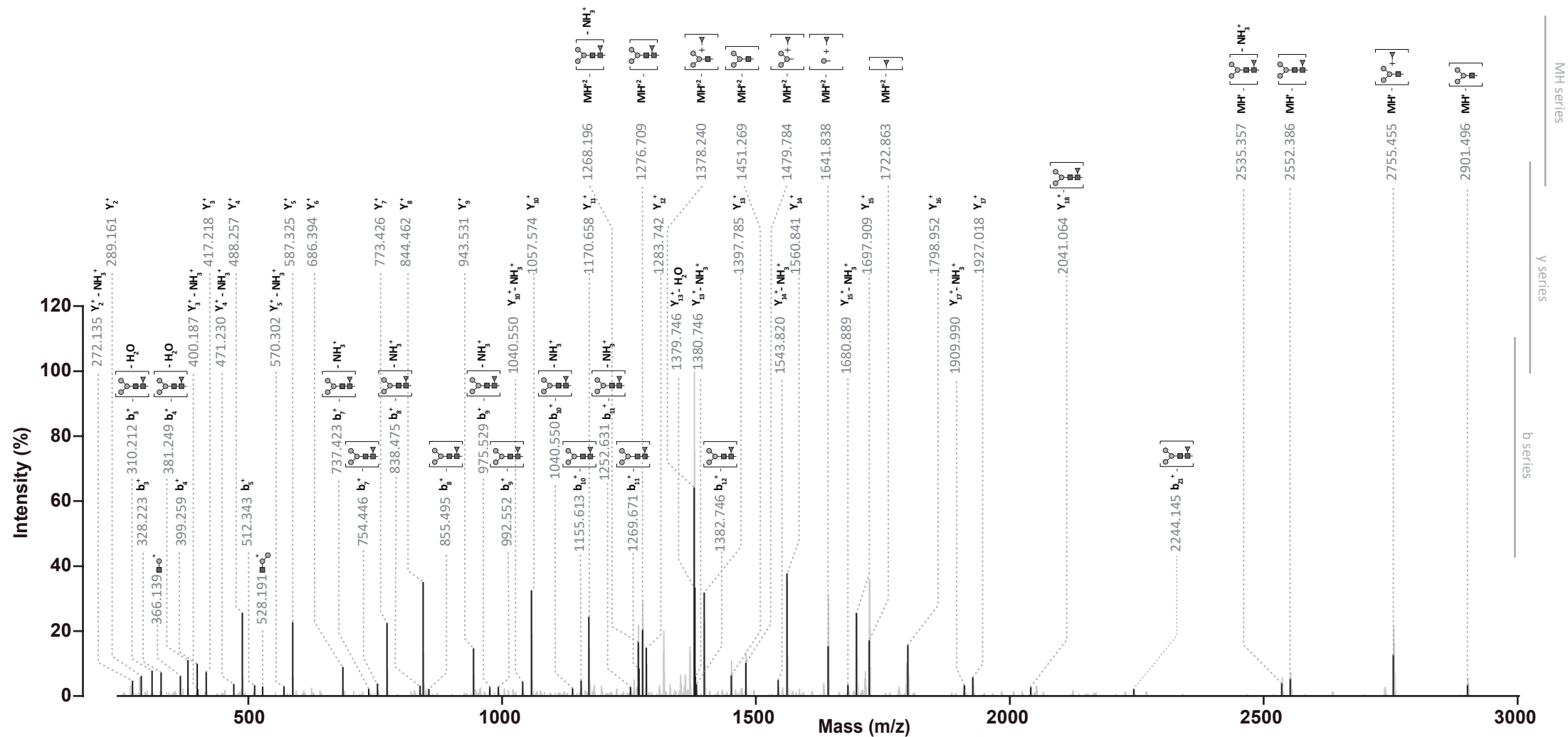

Supplement: Supplementary file 7 — Supplementary Figure S7. [file 41598_2024_62811_MOESM7_ESM.pdf]

Figure S8

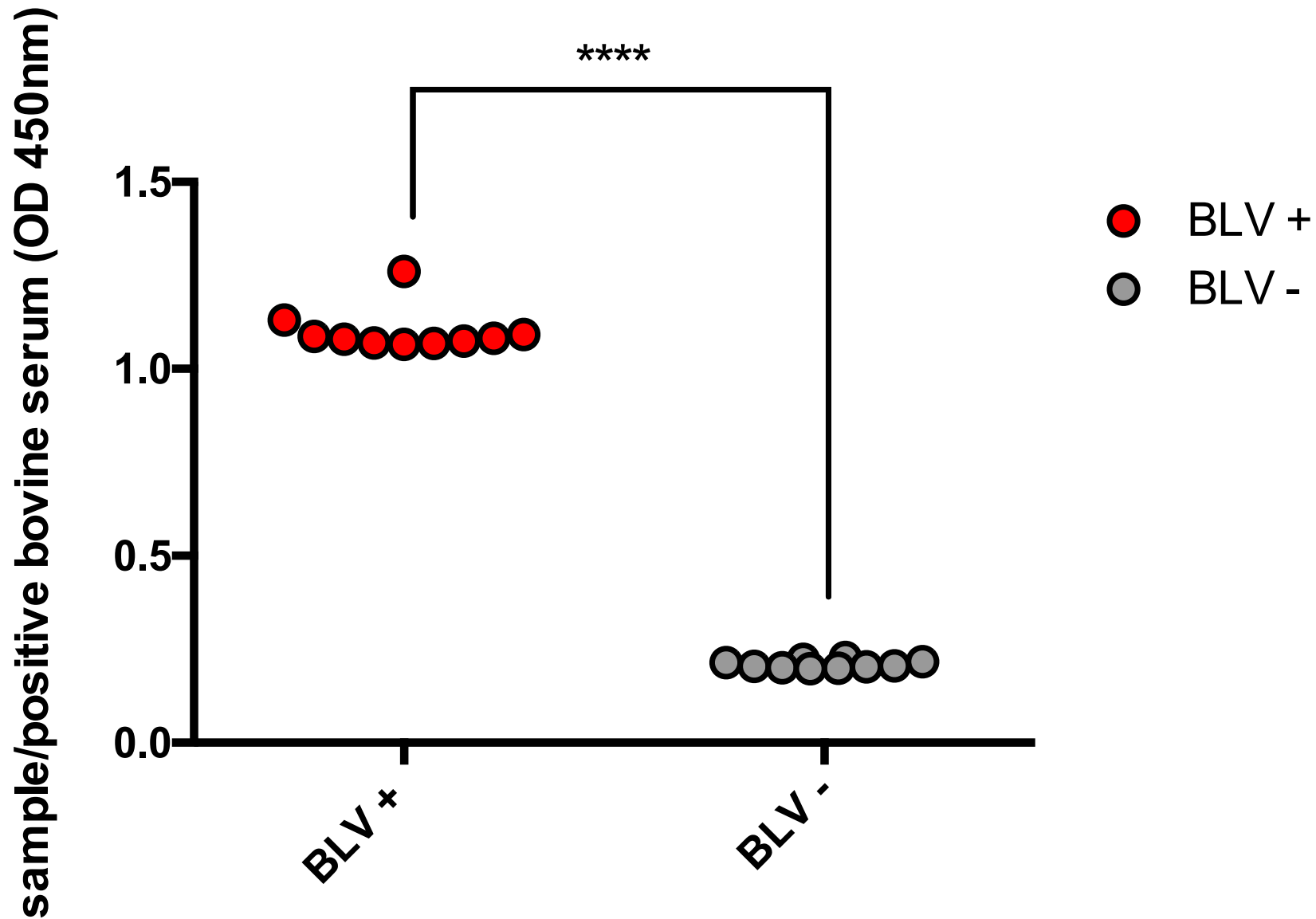

Supplement: Supplementary file 8 — Supplementary Figure S8. [file 41598_2024_62811_MOESM8_ESM.pdf]

**Figure S9**

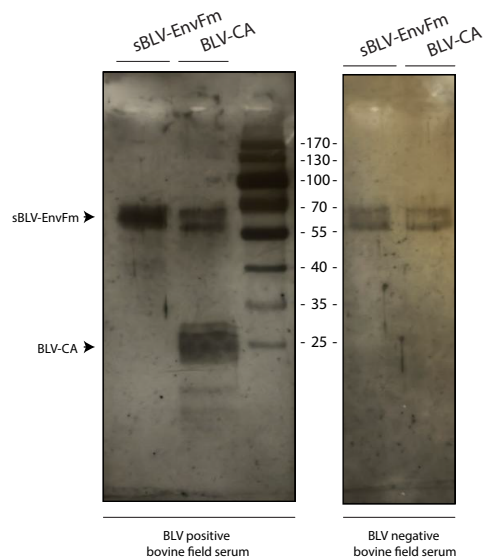

Supplement: Supplementary file 9 — Supplementary Figure S9. [file 41598_2024_62811_MOESM9_ESM.pdf]
