# Supplementary material for: Characterization and application of recombinant Bovine Leukemia Virus Env protein
Source: Sci Rep. 2024 May 28;14:12190. doi: 10.1038/s41598-024-62811-8 (PMC11133380; doi:10.1038/s41598-024-62811-8)
Supplement: Supplementary file 10 — Supplementary Figure S10. [file 41598_2024_62811_MOESM10_ESM.pdf]

Figure S10

33 TWRCSLSLGN QQWMTAYNQE AKFSISIDQI LEAHNQSPFC AKSPRYTLDS
83 VNGYPKIYWP PPQGRRRFGA RAMVTYDCEP RCPYVGADRF DCPHWDNASQ
133 ADQGSFYVNH QILFLHLKQC HGIFTLTWEI WGYDPLITFS LHKIPDPPQP
183 DFPQLNSDWV PSVRSWALLL NQTARAFPDC AICWEPSPPW APEILVYNKT
233 ISSSGPGLAL PDAQIFWVNT SSFNTTQGWH HPSQRLLFNV SQGNALLLPP
283 ISLVNLSTAS SAPPTRVNNS PVAALTLGLA LSVGLTGINV AVSALSHQR
333 TSLIHVLEQD QQRLITAINQ THYNLLNVAS VVAQNRRGLD WLYIRLGFQS
383 LCPTINEPCC FLRIQNDSII RLGDLQPLSQ RVSTDWQWPW NWDLGLTAWV
433 RETIH
