# Supplementary material for: Characterization and application of recombinant Bovine Leukemia Virus Env protein
Source: Sci Rep. 2024 May 28;14:12190. doi: 10.1038/s41598-024-62811-8 (PMC11133380; doi:10.1038/s41598-024-62811-8)

Figure S11

Figure 1b
SEC fractions (-βMe)

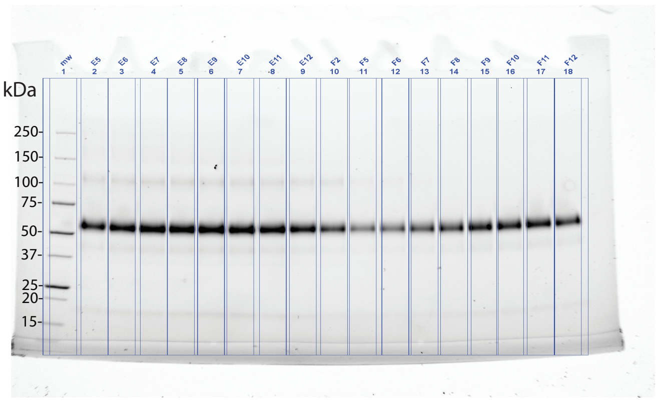

Figure 1d reduced (+βMe)

Figure 1d non-reduced (-βMe)

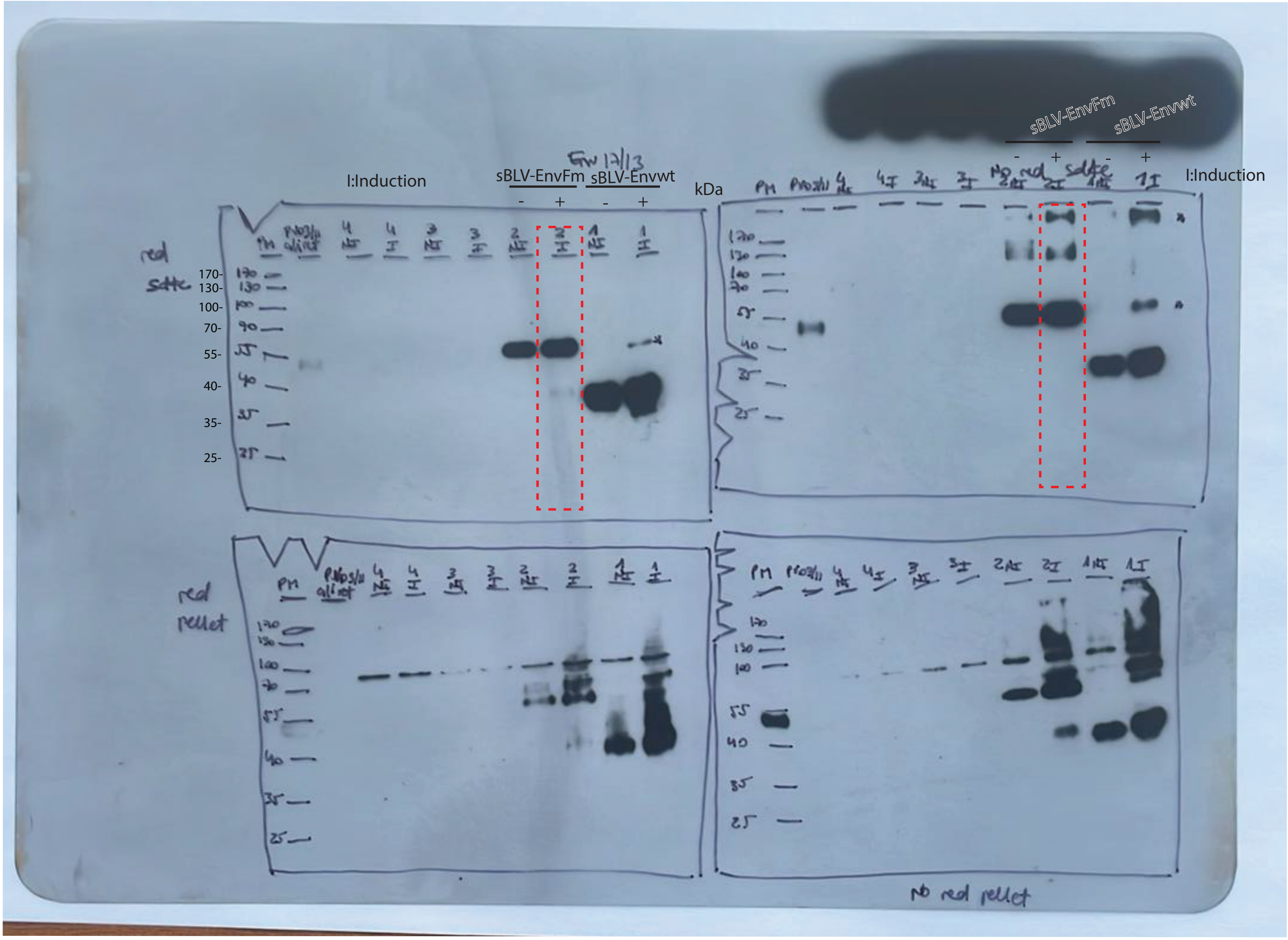

Figure 1e +/- PNGase F

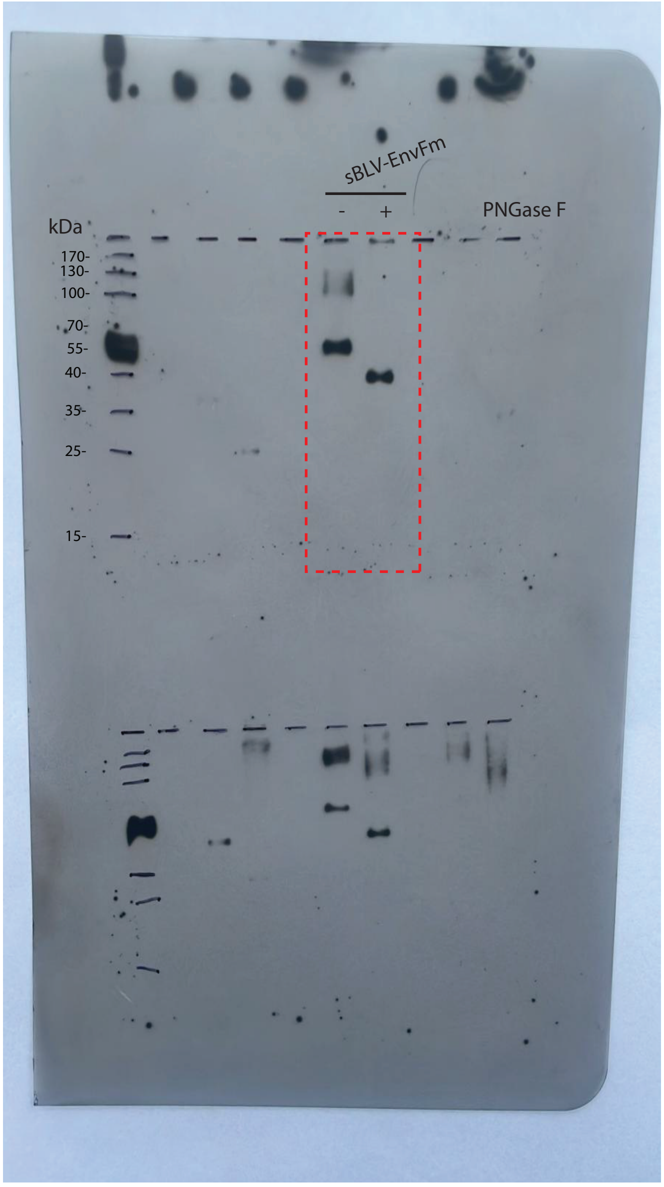

Figure 5a, right

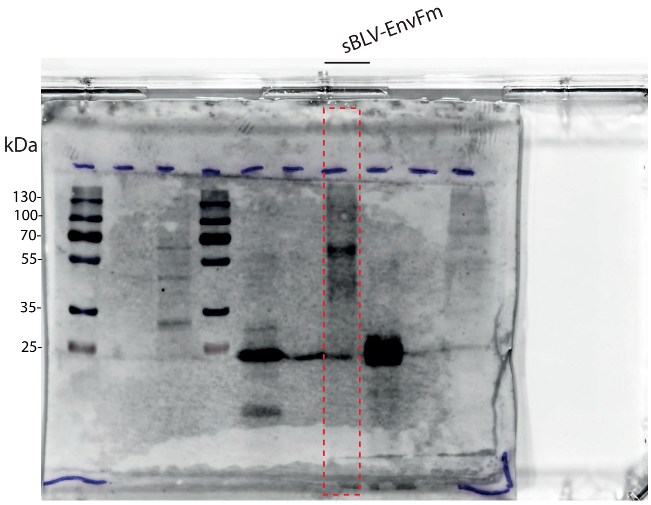

Figure 5b, right

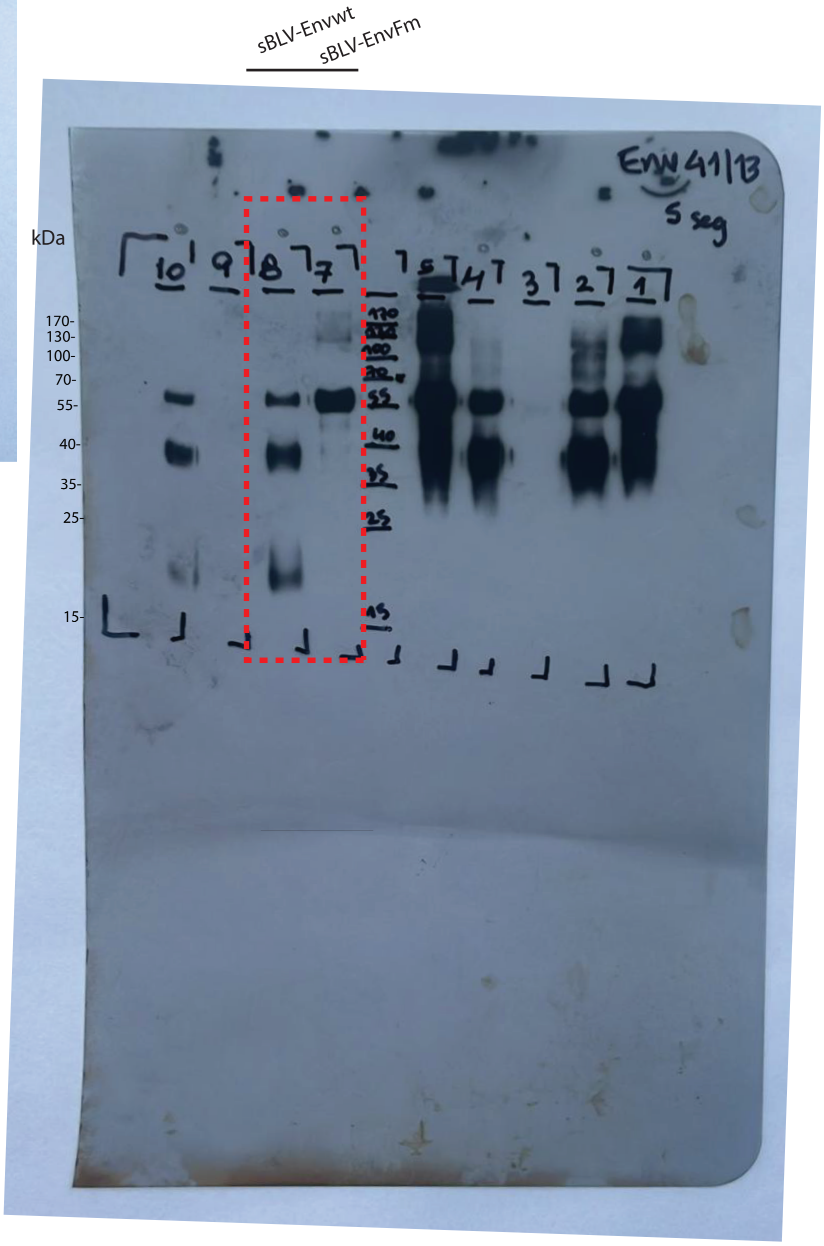

Figure S1

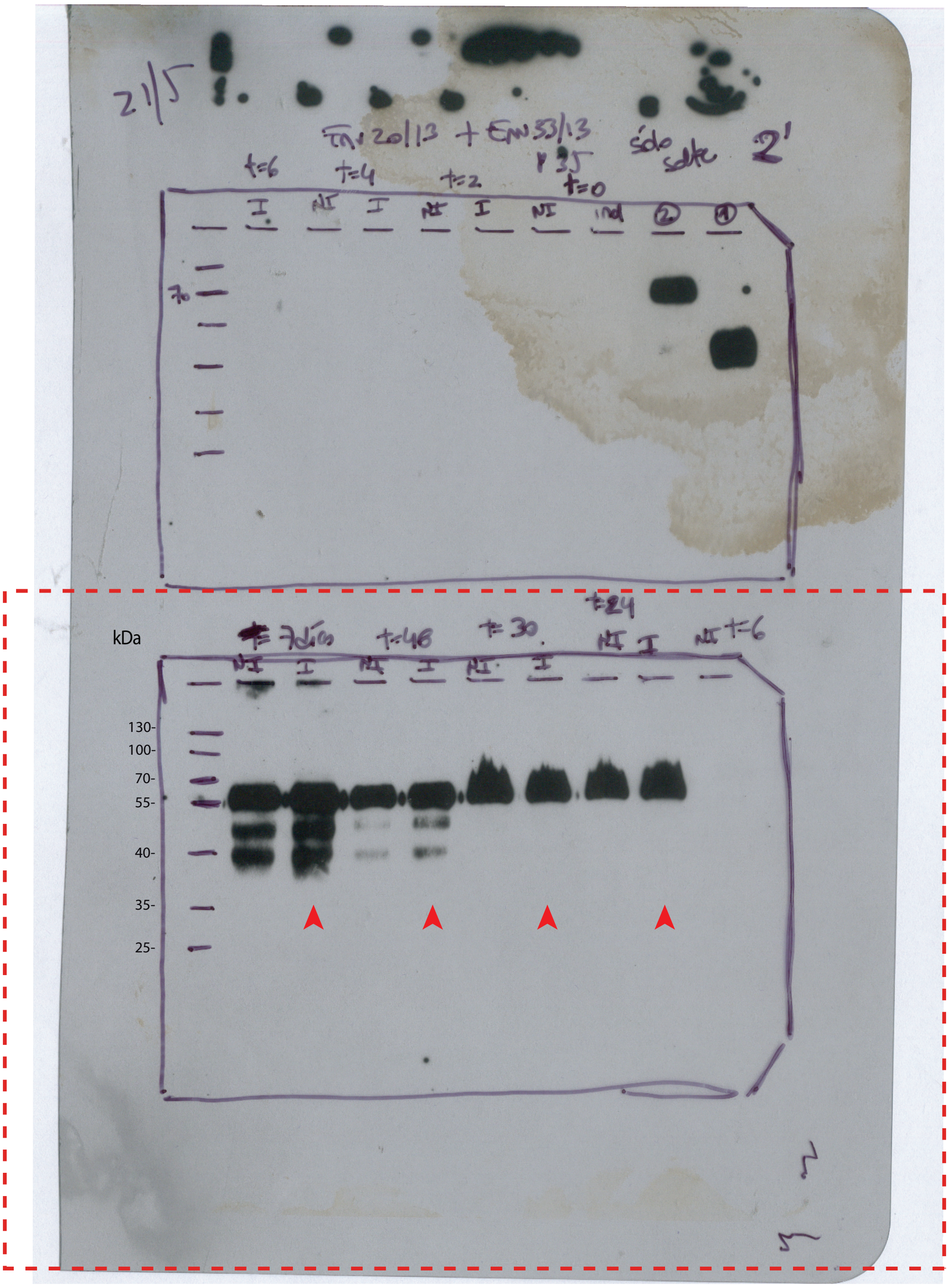

Figure S2b

SEC fractions (-βMe)

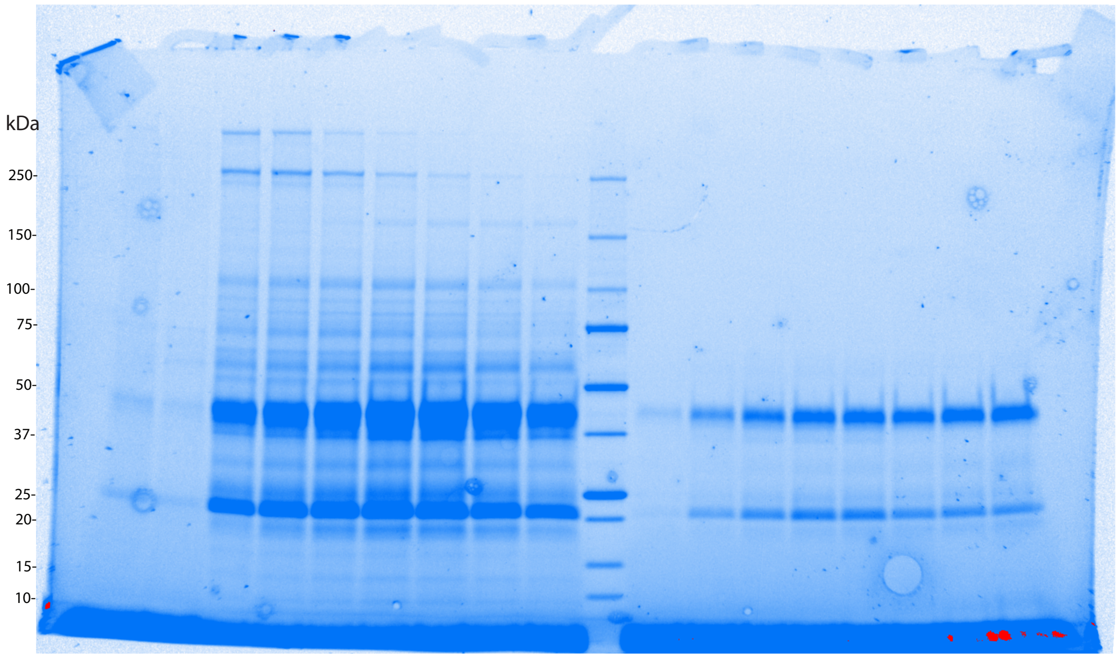

Figure S2c

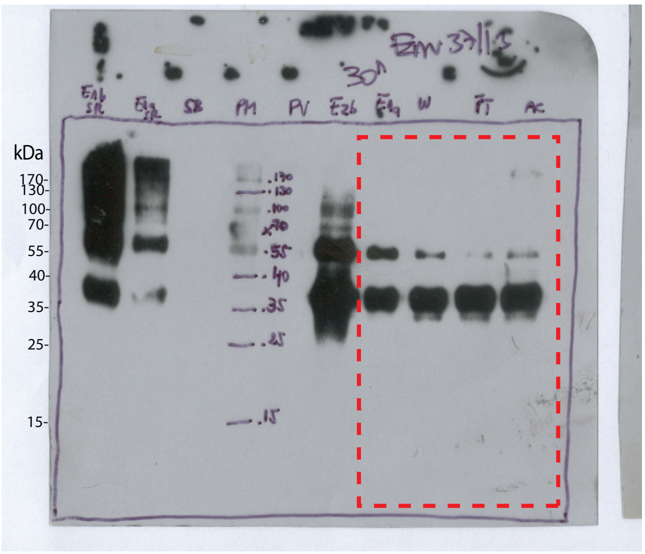

Figure S9

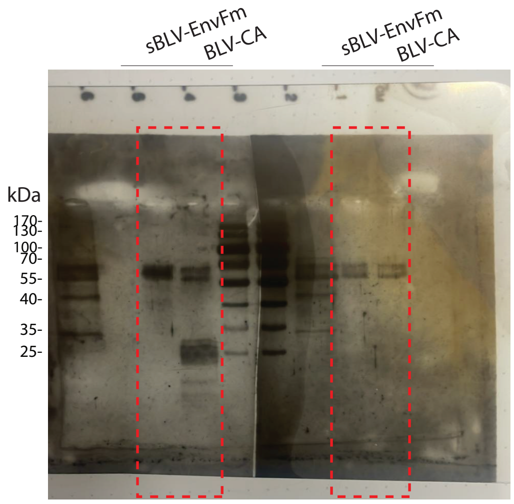

Supplement: Supplementary file 11 — Supplementary Figure S11. [file 41598_2024_62811_MOESM11_ESM.pdf]
