# Supplementary material for: Characterization and application of recombinant Bovine Leukemia Virus Env protein
Source: Sci Rep. 2024 May 28;14:12190. doi: 10.1038/s41598-024-62811-8 (PMC11133380; doi:10.1038/s41598-024-62811-8)
Supplement: Supplementary file 12 — Supplementary Legends. [file 41598_2024_62811_MOESM12_ESM.pdf]

**Supplementary information Tomé-Poderti et al**

**Supplementary Figure Legends**

*Supplementary Figure S1: Expression kinetics of sBLV-EnvFm in supernatants of stable transfected S2 cells after $CdCl_2$ induction.* Time points at 24 hours, 30 hours, 2 days and 7 days post-induction were analyzed by Western Blot using anti-gp51 (BLV2, VMRD Inc., Pullman, WA, USA). Time progression shows sBLV-EnvFm early degradation after 2 days post-induction. A band at 55kDa corresponding to sBLV-EnvFm is shown. Blot was cropped to improve clarity; original blots are presented in Figure S11.

*Supplementary Figure S2: Purification process of recombinant wild type BLV Env protein (sBLV-Envwt).* **(a)** Oligomeric species composition obtained by size-exclusion chromatography (SEC) of StrepTactin-purified sBLV-Env*wt*. High molecular weight and low molecular weight fractions are depicted (dotted grey-boxes). **(b)** Fractions obtained in (a) from the major peak fractions were analyzed by SDS-PAGE under non-reducing conditions. Simple arrowheads show a >37kDa band corresponding to gp51 and a band of 20kDa corresponding to gp30 ectodomain. A 55kDa band corresponding to uncleaved sBLV-Env*wt* is present mainly in high molecular weight fractions (non-cleaved gp51-gp30). **(c)** Western Blot analysis of sBLV-Env*wt* purified by affinity chromatography with anti-gp51 antibody (BLV2, VMRD Inc., Pullman, WA, USA). The horizontal arrow represents the purification flow, with the different fractions obtained in each step (input, flowthrough, wash, elution). Eight micrograms of protein were loaded per lane. Bands at 55 and 37kDa, corresponding to non-

cleaved and furin-processed sBLV-Env*wt* respectively, are present in all the fractions obtained during purification. Gels and blots were cropped to improve clarity; original ones are presented in Figure S11.

*Supplementary Figure S3: Dynamic Light Scattering analysis of sBLV-EnvFm.* The intensity distribution was calculated from three replicates (shown in different colours) and the average hydrodynamic radius ($R_H$) were calculated for each size population (shown in grey above each peak). The overall Polydispersity Index (PdI) is shown in black text in the top right corner of the graph.

*Supplementary Figure S4: NP-HPLC profile of sBLV-EnvFm N-glycans profile after exoglycosidase sequential digestion.* N-glycans released after PNGase F treatment of sBLV-EnvFm were labeled with 2-AB and subjected to normal phase HPLC profile with exoglycosidase digestion in a sequential manner : **(a)** Control **(b)** ABS **(c)** ABS+BKF **(d)** ABS+BKF+BTG **(e)** ABS+BKF+BTG+GUH **(f)** ABS+BKF+BTG+GUH+JBM. Digestion with ABS resulted in negligible modifications of the elution profile, suggesting a marginal or null contribution of sialic acid on glycan moieties (3b). Sequential digestion with BKF caused the loss of the peak at 4,91 GU with a concomitant increase in intensity of the peak at 4,43 GU corresponding to M3 ($Man_3GlcNAc_2$) core (3c), thus suggesting the presence of fucose in the main peak. We confirmed the absence of galactose since no modifications in the elution profile were detected upon digestion with BTG (3d). Similar results were obtained after GUH treatment, suggesting that terminal mannoses imped GUH accessibility to N-acetylglucosamine (GlcNAc) (3e). Finally, digestion with JBM allowed the remotion of

manose residues showing up a peak at 2,65 GU, corresponding to the basic structure $ManGlcNAc_2$ (3f).

ABS : α(2-3,6,8,9)-Arthrobacter ureafaciens sialidase, BKF: Bovine kidney α(1-2,3,4,6)–fucosidase, BTG: bovine testes β(1-3,4) galactosidase, GUH: Streptococcus pneumoniae β-hexosaminidase, JBM: Jack bean α (1-2,3,6)–mannosidase.

G.U. values were derived from a dextran ladder.

mV: millivolts, GU: glucose units, R.time: retention time.

*Supplementary Figure S5: MALDI-ToF MS of 2AB-derivatized N-glycans obtained after PNGase F treatment to confirm glycan structures obtained by NP-HPLC and sequential exoglycosylation assays. Theoretical* m/z values from 2AB-derivatized N-glycans were employed to confirm N-glycan structures using MALDI-ToF MS (positive mode). Differences between m/z experimental values reflected either the gain or the loss of deoxihexose 146Da (fucose) and hexose 162Da (mannose) residues as shown. Theoretical m/z were calculated according to: glycan composition, 2-AB labeling, sodium adduct [M+Na]+ and positive reflector mode using Glycan Mass server (https://glycomass.com/glycan_calculate) and are shown in green boxes next to each glycan structure.

*Supplementary Figure S6: Putative glycosylation sites on the sBLV-EnvFm protein predicted by NetNGlyc 1.0 server (https://services.healthtech.dtu.dk/services/NetNGlyc-1.0/).* The threshold and glycosylation potential are shown. Black and grey bars represent potential N-

glycosylation sites, with strong probability vs threshold limit, respectively. White bars represent non-predicted N-glycosylation sites.

*Supplementary Figure S7: Tandem MS of sBLV-EnvFm glycopeptides obtained by LC-MS for site-specific glycopeptide analysis.* **(a)** Representative tandem MS data of N-glycosylated peptide SWALLLNQTAR. The fragmentation observed from spectra m/z 150-1500 revealed an N-glycan with the composition $Man_3GlcNAc_2$. **(b)** Representative MS/MS spectrum of glycopeptide LITAINQTHYNLLNVASVVAQNR modified with $Man_3GlcNAc_2Fuc$.

Both MS/MS spectra of the (M+2H) 2+ ion of SWALLLNQTAR and LITAINQTHYNLLNVASVVAQNR (doubly charged ions, m/z: 541.5085 and m/z: 897.702, respectively, as calculated with https://prospector.ucsf.edu/prospector/cgi-bin/msform.cgi?form=msproduct) confirmed glycopeptide sequence as well as the N-glycosylation site present in each peptide. The N-terminal and C-terminal fragment ions, including those with partial loss of the glycan structure are labeled. Fragment corresponding b- and y-ions confirming peptides as SWALLLNQTAR or LITAINQTHYNLLNVASVVAQNR are labeled.

*Supplementary Figure S8: ELISA reactivity of bovine field sera against sBLV-EnvFm*. ELISA reactivity of BLV + (red filled dots) and BLV- (grey filled dots) bovine field sera against sBLV-EnvFm is shown (**** $p<0,0001$, unpaired t test). The data was normalized against a positive bovine field serum (used as standard).

*Supplementary Figure S9: Western Blot analysis of sBLV-EnvFm and recombinant BLV capsid protein (CA) with positive and negative BLV bovine field sera.* Positive serum recognizes both sBLV-EnvFm (>55kDa) and BLV-CA (>24kDa) (left) while negative serum does not recognize none of them (right). A non-specific reactive double band was observed ubiquously with all serum samples. BLV-CA protein was expressed as described in Obal *et al*[1]. Total 0.1 micrograms of protein were loaded per lane and bovine sera were diluted 1:2000. Blots were cropped to improve clarity, original blots are presented in Figure S11.

*Supplementary Figure S10: sBLV-EnvFm sequence coverage by mass spectrometry.* sBLV-EnvFm protein sequence coverage (more than 90%) was identified from peptides by MALDI-ToF and nanoLC-MS/MS. Only 3 amino acids (red typeset) could not been identified either by MALDI-ToF or nanoLC-MS/MS. Black typeset indicates non-glycosylated peptides identified by MALDI-ToF and/or nanoLC-MS/MS . Bold black typeset regions indicate glycosylated peptides identified both by MALDI-ToF and nanoLC-MS/MS. Italic bold black typeset indicates glycosylated peptides identified only by MALDI-ToF after PNGase F treatment. Grey bold typeset was used to indicate a glycosylated peptide only identified by nanoLC-MS/MS. Grey bold typeset underlined regions indicate identified glycosylated peptides identified by nanoLC-MS and confirmed by MALDI-ToF (after PNGase F treatment). Black asterisks show putative N-glycosylation sites in the recombinant sBLV-EnvFm as predicted in Figure S5.

As expected for a fully processed protein entering the secretory pathway the BiP sequence was not detected in any case. For this reason BLV Env sequence starts at

amino acid position 33. Modifications included in the searching involved oxidation, carbamidomethylation of cysteines, intact disulfide bonds and N-glycosylation (mannosidic N-glycans).

*Supplementary Figure S11: Original gels and blots employed in the manuscript.* All uncropped images corresponding to electrophoretic gels and blots employed in the main text and in supplementary information are included. Selected areas in each gel employed in the manuscript figures are shown (dashed red box and red arrowheads). Original Figures 5b (right), S1 and S2c were horizontally flipped to better explain the results.

## Materials and Methods

### Oligonucleotide list

| | |
|---|---|
| pT350envopt FOR(33-437) | 5´-ctttgttggcctctcgctcgggagatctacgtggcgctgctcgctgtcgctgggaaatc-3´ |
| pT350 envopt REV (33-437) | 5´-gccttatcgtcatcgtcttcgaagggcccatgaatggtctcgcgcacccaggctgtc-3´ |
| pT350 envopt mutNN FOR | 5´-tcggccccaccaacgcgcgtgaataattcccccgtggccgccctgacactgggcctgg-3´ |

## References


1 Obal, G. *et al.* STRUCTURAL VIROLOGY. Conformational plasticity of a native retroviral capsid revealed by x-ray crystallography. *Science* **349**, 95-98, doi:10.1126/science.aaa5182 (2015).
