# Supplementary material for: Characterization and application of recombinant Bovine Leukemia Virus Env protein
Source: Sci Rep. 2024 May 28;14:12190. doi: 10.1038/s41598-024-62811-8 (PMC11133380; doi:10.1038/s41598-024-62811-8)
Supplement: Supplementary file 13 — Supplementary Table S1. [file 41598_2024_62811_MOESM13_ESM.pdf]

Table S1: N-glycan composition and proposed structures with retention times (min) identified on sBLV-EnvFm N- glycosylation sites. N-glycans released after PNGase F treatment of sBLV-EnvFm were labeled with 2-AB and subjected to normal phase HPLC.

Experimental Retention time (min)	Glucose Unit (GU)	Proposed glycan	GU of proposed glycan	N-glycan abundance (%)
73,985	4,38	M3	4,41±0,13	15,75
78,8-79,9	4,86->4,86	F(6)M3 and/or A1	4,88±0,049 and/or 4,96±0,048	42,20
83,044-85,239	5,15-5,39	M4 and/or A2 and/or F6(A1)	5,28±0,111 and/or 5,43±0,051 and/or 5,44±0,138	2,98
92,274	6,12	M5	6,17±0,033	18,40
99,553	6,98	M6	7,01±0,063	9,24
106,17	7,88	M7	7,85±0,181	6,59
111,851	8,74	M8	8,85±0,169	2,16
