# Supplementary material for: Characterization and application of recombinant Bovine Leukemia Virus Env protein
Source: Sci Rep. 2024 May 28;14:12190. doi: 10.1038/s41598-024-62811-8 (PMC11133380; doi:10.1038/s41598-024-62811-8)
Supplement: Supplementary file 16 — Supplementary Table S4. [file 41598_2024_62811_MOESM16_ESM.pdf]

Table S4: N-glycosylated residues identified by MALDI-ToF upon treatment with PNGaseF.

**Peptides with potential N-glycosylation site**	**Theoretical MH+ (monoisotopic)**	**Experimental MH+ after PNGase (monoisotopic)**	**Δmass**	**N-glycosylation site**
$_{55}$FSISIDQILEAH$\underline{\text{N}}$QSPFCAK$_{74}$	2248.11	2248.07	-0.04	Non-glycosylated
$_{122}$FD**C**PHWD$\underline{\text{N}}$ASQADQGSFYVNHQILFLHLK$_{150}$*	3487.60*	3488.61	1.01	N129
$_{197}$SWALLL$\underline{\text{N}}$QTAR$_{207}$	1272.71	1273.71	1.00	N203
$_{208}$AFPDCAICWEPSPPWAPEILVY$\underline{\text{N}}$K$_{231}$	2744.28	2745.31	1.03	N230
$_{232}$TISSSGPGLALPDAQIFWV$\underline{\text{N}}$TSSF$\underline{\text{N}}$TTQGWHHPSQR$_{267}$	3924.91	3926.90	1.99	N251/N256
$_{268}$LLF$\underline{\text{N}}$VSQGNALLLPPISLV$\underline{\text{N}}$LSTASSAPPTR$_{298}$	3190.78	3192.79	2.01	N271/N287
$_{299}$VNNSPVAALTLGLALSVGLTGINVAVSALSHQR$_{331}$	3242.82	3242.79	-0.03	Non-glycosylated
$_{346}$LITAI$\underline{\text{N}}$QTHYNLLNVASVVAQNR$_{368}$	2552.39	2553.40	1.01	N351
$_{396}$IQ$\underline{\text{N}}$DSIIR$_{403}$	958.53	959.51	0.98	N398

*This peptide was only identified in reduced and alkylated samples containing a modified cysteine (**C**) and this modification was considered for its theoretical MH+ calculation.
$\underline{\text{N}}$: corresponds to predicted N-glycosites. After PNGase treatment, each asparagine residue originally glycosylated (underlined) is converted to aspartic acid, showing a Δmass of +1Da per glycosylated residue.
